# The nutritional impact of replacing dietary meat with meat alternatives in the UK: a modelling analysis using nationally representative data

**DOI:** 10.1017/S0007114521002750

**Published:** 2022-06-14

**Authors:** Dominic N. Farsi, Dinithi Uthumange, Jose Munoz Munoz, Daniel M. Commane

**Affiliations:** Department of Applied Sciences, Northumbria University, Newcastle upon Tyne, NE1 8ST, UK

**Keywords:** Meat replacement, meat alternatives, Nutritional intake, Nutritional requirements, Public health

## Abstract

Dietary patterns high in meat compromise both planetary and human health. Meat alternatives may help to facilitate meat reduction; however, the nutritional implications of displacing meat with meat alternatives does not appear to have been evaluated. Here, the ninth cycle of the National Diet and Nutrition Survey was used as the basis of models to assess the effect of meat substitution on nutritional intake. We implemented three models; model 1 replaced 25 %, 50 %, 75 % or 100 % of the current meat intake with a weighted mean of meat alternatives within the UK market. Model 2 compared different ingredient categories of meat alternative; vegetable, mycoprotein, a combination of bean and pea, tofu, nut and soya. Model 3 compared fortified *v*. unfortified meat alternatives. The models elicited significant shifts in nutrients. Overall, carbohydrate, fibre, sugars and Na increased, whereas reductions were found for protein, total and saturated fat, Fe and B_12_. Greatest effects were seen for vegetable-based (+24·63g/d carbohydrates), mycoprotein-based (–6·12g/d total fat), nut-based (–19·79g/d protein, +10·23g/d fibre; −4·80g/d saturated fat, +7·44g/d sugars), soya-based (+495·98mg/d Na) and tofu-based (+7·63mg/d Fe, −2·02μg/d B_12_). Our results suggest that meat alternatives can be a healthful replacement for meat if chosen correctly. Consumers should choose meat alternatives low in Na and sugar, high in fibre, protein and with high micronutrient density, to avoid compromising nutritional intake if reducing meat intake. Manufacturers and policy makers should consider fortification of meat alternatives with nutrients such as Fe and B_12_ and focus on reducing Na and sugar content.

Globally, annual per capita meat consumption has increased by 20 kg since 1961^(1)^. This sector of our food system contributes substantially to greenhouse gas emissions^(2)^, depletes natural resources^(3–5)^ and compromises animal welfare. In the UK, meat can be a valuable source of nutrients; however, overconsumption is associated with an increased risk of CVD, type 2 diabetes^(6–8)^ and cancer^(9–16)^. Reducing meat consumption may therefore promote human health, protect animal welfare and benefit the environment.

Changing dietary behaviours to elicit a transition towards alternative protein sources is challenging in part owing to strongly held taste preferences, culinary traditions and social and cultural norms^(17)^. Meat alternatives, resembling meat in appearance, preparation and eating experience, could therefore play an important role in enabling dietary change^(18)^. Integration of meat alternatives into the diet is subtle and does not require an overhaul of food consumption and meal patterns^(19–21)^. Although meat alternatives have been available for years, their popularity with consumers has increased rapidly in recent times; due in part to producers better simulating the taste, texture and functionality of traditional meat products. There has also been an increase in the direction of marketing towards meat-eating consumers, rather than just vegans and vegetarians^(22)^.

The rapid increase in the consumption of meat alternatives has raised concerns regarding their overall healthfulness and the potential displacement of valuable nutrients. The developing meat alternative market includes an array of food products with variable nutritional quality, some of which might be considered ultra-processed^(23)^. If these foods are to play a role in reducing meat consumption at a population level, it is essential that any population level nutritional benefits and consequences are identified early and actions taken to mitigate undesirable effects.

Here, we model the potential nutritional implications of meat replacement by using the ninth cycle (years 2016–2017) of the nationally representative UK National Diet and Nutrition Survey rolling program (NDNS RP). We present the nutritional implications of complete and partial meat substitution, using models that either assume replacement of meat with a range of alternative products, based on current UK purchasing data, or models that assume replacement of meat with specific meat alternative ingredient categories. To our understanding, this is the first study to evaluate the nutritional implications of such dietary-replacement scenarios.

## Methods

### Study population

Dietary intake data for the UK population were drawn from the ninth cycle of the NDNS RP. The NDNS RP is a Public Health England and Food Standards Agency funded survey of the food consumption, nutrient intake and nutritional status of people aged 1·5 years and older living in private households in the UK and is representative of the UK population^(24)^. Detailed descriptions of the NDNS RP survey design and sampling methods can be found elsewhere^(25–27)^. The 2016–2017 survey cycle year used in the present modelling analysis included 1253 participants aged 1·5–95 years.

### Dietary records

The NDNS RP collected habitual dietary data using 4-d estimated food diaries^(24–26)^. Data from food diaries have been aggregated for each respondent to provide publicly available daily averages of self-reported food and nutrient intake. For this analysis, we excluded data sets for participants with extreme reported energy intakes (<2092 kJ – >20920 kJ). Data sets from subjects who self-reported abstaining from meat, or with dietary data devoid of any meat, were also excluded. A common occurrence in the collection of habitual dietary intake data is underreporting of energy intake^(28)^, which the NDNS RP data drawn upon here corroborates. There are different methods to approach underreporting^(29)^; in the present analysis, we adjusted nutrients to age and gender-specific estimated average requirements for energy.

Sub-analyses were performed by stratifying by sex and age group, using the same stratification system as the NDNS RP (male and female; 4–10 years, 11–18 years and 19–64 years, over 65 years). Children in 1·5–3 years subgroups were not included as nutrient requirements are specific for each age in this sub-population. In addition, dietary patterns at this stage in life may not include substantial amounts of solid food such as meat. This left a total of 1110 respondents for inclusion in the analysis.

### Self-reported nutritional intake from meat

The current level of meat consumption in the UK was estimated from the NDNS data set. Briefly, the NDNS RP reports meat intake in grams per day broken down by type (i.e. ‘beef, lamb, pork, offal etc’), We allocated these categories into food type groups; ‘red meat; ‘processed red meat’, ‘white meat’, ‘processed white meat’ and ‘seafood’. In this analysis seafood was included with meat as a source of animal flesh. We note that the UK dietary guidelines encourage a population level increase in fish consumption as a source of omega 3 fatty acids and other nutrients, however, most UK vegetarians describe themselves as lacto-ovo-vegetarian or vegan, and only a minority describe themselves as pescatarian.

The allocation and stratification of animal meat within the NDNS data set is described in Table 1.

**Table 1.** NDNS food groups used to calculate self-reported meat intake

	Meat subtype	NDNS category*	Description of category
	Red meat	“beefg”	Amount of beef consumed (g)
		“lambg”	Amount of lamb consumed (g)
		“porkg”	Amount of pork consumed (g)
		“offalg”	Amount of offal consumed (g)
		“liverdishes”	Amount of liver consumed (g)
		“otherredmeatg”	Amount of other red meat consumed (g)
Total Meat	Processed red meat	“burgersg”	Amount of burger consumed (g)
		“sausagesg”	Amount of sausages consumed (g)
		“baconandham”	Amount of bacon and/or ham consumed (g)
		“burgersandkebabs”	Amount of burger and/or kebab consumed (g)
		“processedredmeatg”	Amount of other processed red meat consumed (g)
	White meat	“poultryg”	Amount of poultry consumed (g)
		“gamebirdsg”	Amount of game consumed (g)
	Processed white meat	“coatedchicken”	Amount of coated chicken products consumed (g)
		“processedpoultryg”	Amount of processed poultry consumed (g)
	Seafood	“whitefishg”	Amount of white fish consumed (g)
		“oilyfishg”	Amount of oily fish consumed (g)
		“cannedtunag”	Amount of canned tuna consumed (g)
		“shellfishg”	Amount of shellfish consumed (g)
		“whitefishcoatedorfried”	Amount of white fish (coated or fried) consumed (g)

* As displayed in NDNS data set.

The total nutritional contribution of meat to the UK diet was estimated by combining the intake data with the nutritional composition data from the McCance and Widdowson composition of food tables^(30)^. Briefly, an aggregate quantity of nutrient, per gram, for each of the meat subtypes was multiplied by the intake in grams per day, for each respondent. The totality of these nutrition scores by subtype was then used to estimate the total nutritional value of meat products to the diet for each individual respondent.

### Nutritional content of meat alternatives

Products were identified through product searches on UK supermarket websites using the key words, ‘meat alternatives’, ‘meat substitutes’, ‘meat-free’, ‘plant-based’, ‘vegan’ and ‘vegetarian’, to ensure a wide capture of meat alternative products. Following data collection, the products that met these criteria were grouped into ingredient categories (Supplementary Table S1).

The nutritional profile of meat alternatives was calculated based on product labels, information from supermarket and manufacturer websites, as well as searching nutrition databases for products. Where nutrient data was not available for a product, manufacturers were contacted to request further details. This data was used to estimate the aggregate nutritional composition per gram for each of the meat alternative ingredient categories, based on the mean nutritional composition of the individual products included in each respective category.

There was incomplete availability of nutritional data across the product categories for many of the micronutrients including I, Zn, and Se. There was available nutritional data for the following nutrients: carbohydrate, protein, total fat, saturated fat, fibre, sugar, Na, Fe and B_12_. We deemed the data on these nutrients of good quality for inclusion in subsequent analysis

### Meat-replacement scenarios

Here, we assessed the nutritional implications of a set of scenarios for the widespread replacement of meat with meat alternatives in the UK population (Table 2).

**Table 2.** Meat-replacement models implemented in the present modelling analysis

Replacement Scenario Models		Description of model
Progressive (Model 1)	MA-25	Partial replacement substituting 25 % of the current self-reported meat intake with a composite of meat alternatives.
	MA-50	Partial replacement substituting 50 % of the current self-reported meat intake with a composite of meat alternatives.
	MA-75	Partial replacement substituting 75 % of the current self-reported meat intake with a composite of meat alternatives.
	MA-100	Full replacement substituting 100 % of the current self-reported meat intake with a composite of meat alternatives.
Ingredient (Model 2)	Vegetable	Full replacement substituting 100 % of the current self-reported meat intake with meat alternatives produced predominantly from vegetables.
	Mycoprotein	Full replacement substituting 100 % of the current self-reported meat intake with meat alternatives produced predominantly from mycoprotein.
	Legume	Full replacement substituting 100 % of the current self-reported meat intake with meat alternatives produced predominantly from bean and pea.
	Tofu	Full replacement substituting 100 % of the current self-reported meat intake with meat alternatives produced from tofu.
	Nut	Full replacement substituting 100 % of the current self-reported meat intake with meat alternatives produced predominantly from nut.
	Soya	Full replacement substituting 100 % of the current self-reported meat intake with meat alternatives produced predominantly from soya.
Fortification (Model 3) *	Fortified	Full replacement substituting 100 % of the current self-reported meat intake with meat alternatives that have been fortified with nutrients.
	Unfortified	Full replacement substituting 100 % of the current self-reported meat intake with meat alternatives that have not been fortified with nutrients.

*Products identified as fortified if 1) explicitly stated nutrient fortification had been applied during manufacturing process; 2) isolated nutrients included in ingredient list. Products not meeting 1) or 2) identified as unfortified.

#### Model 1: the effect of meat reduction and replacement with meat alternatives

In the first replacement model, we generated a weighted mean of the nutritional quality of meat alternatives based on the current market share for meat alternative products in the UK; vegetable 23 %, mycoprotein 18 %, bean 17 %, soya 15 %, nut 14 % and tofu 13 %^(31)^.

We then looked at the effect of progressively reducing the amount of meat in each respondent’s diet by 25 % (MA-25), 50 % (MA-50), 75 % (MA-75) and 100 % (MA-100) and replacing the removed nutrients with the weighted nutritional score of the meat alternatives on a per weight basis (Table 2).

#### Model 2: a comparison of the effects of replacing nutrients from meat across specific ingredient categories of meat alternative

In a second model, we compared the nutritional impact of replacing meat with meat alternatives produced from a particular category of ingredient. Specifically, we replaced the total amount of meat in each individual respondent’s diet with vegetable-based (Vegetable), mycoprotein-based (Mycoprotein), a combination of bean and pea-based (Legume), tofu-based (Tofu), nut-based (Nut) and soya-based (Soya) meat alternatives (Table 2).

#### Model 3: substitution with fortified *v*. unfortified meat alternatives

Within the food products identified, 14 % were fortified with either Fe, B_12_, or both. Taking this into account, in a third and final model, we compared the effects of replacing the total amount of meat in each person’s diet with either fortified meat alternatives (Fortified) or unfortified meat alternatives (Unfortified) (Table 2).

We implemented each replacement strategy by removing the nutrient contribution of meat from each individual respondents’ total nutritional intake, followed by replacing this with the nutritional data for the equivalent weight of meat alternatives. We adjusted the final total nutritional intake after substitution to match the energy content of each participant’s diet.

### Comparators and subjective model corrections

Due to the variable quality of available nutritional data and the relevance to public health, we have elected in this analysis to focus on the macronutrients, protein, fat and carbohydrate, as well as saturated fat, total sugars and Na due to public health guidance to limit their intake^(32,33)^. In addition, fibre was selected as authoritative bodies advise an intake of 30 g/d for the adult population^(33)^; which many fail to achieve^(34)^. We hypothesised that fibre would increase in the replacement models as a result of the higher fibre content of meat alternatives compared with meat^(35)^. We also assessed influences on Fe and B_12_, as the intake of these nutrients may be inadequate when transitioning to a dietary pattern devoid of animal based foods^(36)^.

We analysed the nutritional adequacy of the meat-replacement models by comparing the nutrient values to the dietary reference values (DRV) for Food Energy and Nutrients for the UK^(32,33,37)^. DRV comprise a series of estimates of the amount of energy and nutrients needed by different groups of healthy people in the UK population. In accordance with the DRV, we used the estimated average requirements as a comparator for total energy, whereas for protein, Na, Fe and B_12_, the reference nutrient intakes (RNI) were used. For fat and carbohydrates (including total sugars, saturated fat and fibre), we used the UK DRV which for total fat (35 %), saturated fat (11 %) and total carbohydrates (50 %) are given as a percentage of daily energy intake^(32,33,37)^. The current recommendations for protein is to intake 0·75 g/kg bodyweight^(33,37)^, as the data set in the current analysis contains a diverse age range which will also comprise different body types, a protein target of making up the remaining 15 % of total energy intake after the contributions of fat (35 %) and carbohydrates (50 %) was employed instead.

Since the recommendations differ by age and sex, we calculated the average values for nutrients across the population subgroups (male and female; 4–10 years, 11–18 years, 19–64 years and over 65 years). We also incorporated a group that represented the total number of participants (both male and female, ages 4 years and above) and averaged all DRV categories to obtain a population average. This group would act as the representative of the UK population and used in the main analysis.

### Statistical analysis

Differences in nutrient intake between the meat-replacement models and the current intake were compared using linear models with nutrients as the dependent variable and the replacement models as the independent variable, using the current intake as the reference. The differences in nutrient intake between all scenarios (current and replacement models) with reference to DRV were then compared using linear models in the same manner but including the current intake with the replacement models as the independent variable and then using DRV used as the reference. To assess the nutrient intake between meat-replacement models, an ANOVA was implemented, using an *α* = 0·05, followed by a post hoc Tukey test to determine the significance of differences between models. All analyses were performed using R statistical software (version 4.0.0)^(38)^.

## Results

### Self-reported meat intake

The average self-reported intake of both total meat and meat subgroup for the total population, as well separate population subgroups, is shown in Table 3. For the total population, the self-reported average meat intake was 132·25 g/d (95 % CI, 128·02–136·48 g/d). Processed meat was the largest contributor to total meat intake (35·98 %), followed by white meat (27·22), red meat (19·44 %) and seafood (13·36 %), with processed white meat contributing the lowest (3·99 %). Males aged 19–64 years consumed the greatest amount of meat (mean, 177·55 g/d; 95 % CI, 165·33–189·77 g/d), while girls aged 4–10 years consumed the least amount of meat (mean, 98·75 g/d; 95 % CI, 91·28–106·23 g/d).

**Table 3.** Self-Reported meat intake (total meat and meat subtype) stratified by age and gender (Mean values and 95 % confidence intervals)

Subgroup		Total meat, g/d		Red meat, g/da		Contribution to total meat, %		Processed meat, g/d		Contribution to total meat, %		White meat, g/d		Contribution to total meat, %		Processed white meat, g/d		Contribution to total meat, %		Seafood, g/d	Contribution to total meat, %	
	Mean	95 % CI	Mean	95 % CI	Mean	95 % CI	Mean	95 % CI	Mean	95 % CI	Mean	95 % CI	Mean	95 % CI	Mean	95 % CI	Mean	95 % CI	Mean	95 % CI	Mean	95 % CI
Male, 4 − 10years	106·01	96·61, 115·42	14·76	11·74, 17·77	13·92	12·16, 15·39	47·66	41·19, 54·13	44·95	42·63, 46·89	26·27	22·30, 30·24	24·78	23·08, 26·20	6·41	4·88, 7·95	6·05	5·05, 6·89	10·92	8·64, 13·20	10·30	8·95, 11·43
Female, 4 − 10years	98·75	91·28, 106·23	13·72	10·92, 16·51	13·89	11·97, 15·54	40·97	34·84, 47·11	41·49	38·17, 44·35	24·47	20·88, 28·07	24·78	22·87, 26·42	6·88	5·26, 8·49	6·96	5·77, 7·99	12·71	10·20, 15·22	12·87	11·18, 14·33
Male, 11 − 18 years	146·71	134·32, 159·10	21·4	16·47, 26·32	14·59	12·26, 16·54	60·37	50·55, 70·18	41·15	37·63, 44·11	40·79	33·57, 48·01	27·8	24·99, 30·17	9·14	5·71, 12·57	6·23	4·25, 7·90	15·01	10·57, 19·46	10·23	7·87, 12·23
Female, 11 − 18 years	131·36	117·86, 144·87	19·87	15·51, 24·23	15·12	13·16, 16·72	50·21	40·47, 59·94	38·22	34·34, 41·38	39·70	34·19, 45·21	30·22	29·01, 31·21	9·41	6·75, 12·07	7·16	5·73, 8·33	12·18	9·25, 15·10	9·27	7·85, 10·42
Male, 19 − 64 years	177·55	165·33, 189·77	40·07	34·21, 45·93	22·57	20·69, 24·20	62·46	53·53, 71·40	35·18	32·38, 37·62	50·05	43·67, 56·44	28·19	26·41, 29·74	5·04	3·34, 6·74	2·84	2·02, 3·55	19·92	16·09, 23·75	11·22	9·73, 12·51
Female, 19 − 64 years	127·84	119·63, 136·06	26·33	22·44, 30·21	20·59	18·76, 22·20	38·94	33·29, 44·59	30·46	27·83, 32·77	39·41	34·79, 44·02	30·83	29·09, 32·36	3·31	2·16, 4·46	2·59	1·81, 3·28	19·86	16·76, 22·95	15·53	14·01, 16·87
Male, 65 years +	151·01	133·53, 168·49	42·22	33·65, 50·80	27·96	25·20, 30·15	52·64	40·51, 64·77	34·86	30·34, 38·44	29·01	19·20, 38·82	19·21	14·38, 23·04	1·18	0·04, 2·41	0·78	0·03, 1·43	25·95	20·12, 31·78	17·18	15·07, 18·86
Female, 65 years +	103·97	94·29, 113·66	26·61	20·68, 32·54	25·60	21·94, 28·63	27·29	21·29, 33·30	26·25	22·58, 29·30	21·98	16·59, 27·36	21·14	17·59, 24·08	0·80	0·09, 1·51	0·77	0·10, 1·33	27·29	21·66, 32·92	26·25	22·97, 28·96
Total Population	132·25	128·02, 136·48	25·72	23·88, 27·55	19·44	18·65, 20·19	47·58	44·68, 50·49	35·98	34·90, 36·99	36·00	33·88, 38·12	27·22	26·46, 27·93	5·28	4·61, 5·95	3·99	3·60, 4·36	17·67	16·31, 19·04	13·36	12·74, 13·95

### Projected changes in nutritional intake

Overall, the implemented meat-replacement scenarios elicited many differences in the nutritional intake. The current nutrient intake for the total population and for each replacement model (MA-25, MA-50, MA-75, MA-100, Vegetable, Mycoprotein, Legume, Tofu, Nut, Soya, Fortified, Unfortified) is included in Table 4, while the projected differences for each replacement model in comparison to the current intake are included in Table 5.

**Table 4.** Mean nutrient intake of total population (*n* 1110) for current and replacement models, with reference to dietary reference values

			Progressive model				Ingredient model						Fortification model	
Nutrient	DRV*	Current	MA-25	MA-50	MA-75	MA-100	Vegetable	Mycoprotein	Legume	Tofu	Nut	Soya	Fortified	Unfortified
Energy, kJ/d	8995.60	–	–	–	–	–	–	–	–	–	–	–	–	–
Carbohydrate, g/d†	268·80	270·77	275·16	279·78	284·65	289·80	295·39	293·17	290·68	279·77	282·24	285·33	282·01	294·03
Protein, g/d†	80·60	86·67	83·27	79·68	75·90	71·90	67·34	77·86	71·24	81·80	66·88	79·48	83·58	68·49
Fat, g/d	83·60	81·97	81·19	80·38	79·51	78·60	77·58	75·85	78·48	79·60	83·35	77·26	77·08	77·78
Fibre, g/d	30	21·86	23·74	25·71	27·80	30·00	30·59	30·57	28·66	25·00	32·10	28·12	28·94	30·23
Sugars, g/d	90	110·52	111·48	112·49	113·56	114·68	114·94	113·30	113·93	112·66	117·96	112·71	112·20	114·63
Saturated fat, g/d	26·30	30·96	30·05	29·08	28·07	27·00	27·13	27·31	27·23	26·63	26·16	27·27	27·66	27·02
Na, mg/d	2400	2413·80	2466·37	2521·74	2580·14	2641·82	2720·90	2726·12	2690·65	2510·20	2289·49	2909·77	2896·42	2774·88
Fe, mg/d	8·7/14·8‡	13·65	13·59	13·52	13·45	13·38	13·17	12·68	13·26	21·28	12·74	13·61	16·77	13·10
B_12_, μg/d	1·5	7·47	7·07	6·65	6·21	5·74	5·72	5·98	5·77	5·45	5·59	5·71	7·14	5·68

DRV, dietary reference value; EAR, estimated average requirement; RNI, reference nutrient intake; MA-25, replacement model substituting 25 % of self-reported meat intake with composite of meat alternatives; MA-50, replacement model substituting 50 % of self-reported meat intake with composite of meat alternatives; MA-75, replacement model substituting 75 % of self-reported meat intake with composite of meat alternatives; MA-100, replacement model substituting 100 % of self-reported meat intake with composite of meat alternatives.

Colours: Green, meeting DRV. Red, not meeting DRV. Grey, not meeting 15 % protein and/or 50 % carbohydrate target elected in the current analysis. Blue, not meeting higher RNI for iron.

*DRV values correspond to EAR for energy; RNI for protein, Na, Fe, B_12_; DRV for carbohydrate, fat, fibre, sugars, saturated fat.

† Protein target elected as 15 % total energy intake and carbohydrate as 50 % total energy intake in the current analysis.

‡ Iron RNI for females aged 11–50 years.

**Table 5.** Projected differences from current intake for meat alternatives across the total population (*n* 1110)

Progressive Model													
	Current	MA-25	*P*	MA-50	*P*	MA-75	*P*	MA-100	*P*				
Energy, kJ/d	8995.60	–	–	–	–	–	–	–	–				
Carbohydrate, g/d	270·77	+4·39	0·25	+9·01	0·018*	+13·89	<0·001*	+19·04	<0·001*				
Protein, g/d	86·67	–3·40	0·002*	–6·99	<0·001*	–10·77	<0·001*	–14·77	<0·001*				
Fat, g/d	81·97	–0·78	0·55	–1·60	0·22	–2·46	0·06	–3·37	0·009*				
Fibre, g/d	21·86	+1·88	<0·001*	+3·85	<0·001*	+5·93	<0·001*	+8·13	<0·001*				
Sugars, g/d	110·52	+0·96	0·66	+1·97	0·36	+3·04	0·16	+4·16	0·05*				
Saturated fat, g/d	30·96	–0·91	0·09	–1·88	0·001*	–2·89	<0·001*	–3·96	<0·001*				
Na, mg/d	2413·80	+52·57	0·21	+107·94	0·01	+166·34	<0·001*	+228·03	<0·001*				
Fe, mg/d	13·65	–0·06	0·93	–0·13	0·85	–0·20	0·78	–0·27	0·70				
B_12_, μg/d	7·47	–0·40	0·66	–0·82	0·37	–1·26	0·17	–1·73	0·06				

Ingredient Model													
	Current	Vegetable	*P*	Mycoprotein	*P*	Legume	*P*	Tofu	*P*	Nut	*P*	Soya	*P*
Energy, kJ/d	8995.60			–	–	–	–					–	–
Carbohydrate, g/d	270·77	+24·63	<0·001*	+22·40	<0·001*	+19·92	<0·001*	+9·00	0·018*	+11·48	0·003*	+14·56	<0·001*
Protein, g/d	86·67	–19·33	<0·001*	–8·82	<0·001*	–15·43	<0·001*	–4·87	<0·001*	–19·79	<0·001*	–7·19	<0·001*
Fat, g/d	81·97	–4·39	0·001*	–6·12	<0·001*	–3·49	0·007*	–2·37	0·07	+1·38	0·29	–4·71	<0·001*
Fibre, g/d	21·86	+8·73	<0·001*	+8·71	<0·001*	+6·79	<0·001*	+3·14	<0·001*	+10·23	<0·001*	+6·25	<0·001*
Sugars, g/d	110·52	+4·43	0·04*	+2·78	0·20	+3·42	0·11	+2·15	0·32	+7·44	0·001*	+2·20	0·31
Saturated fat, g/d	30·96	–3·83	<0·001*	–3·65	<0·001*	–3·73	<0·001*	–4·33	<0·001*	–4·80	<0·001*	−3·69	<0·001*
Na, mg/d	2413·80	+307·10	<0·001*	+312·32	<0·001*	+276·85	<0·001*	+96·40	0·02*	−124·30	0·003*	+495·98	<0·001*
Fe, mg/d	13·65	−0·48	0·49	−0·97	0·16	−0·39	0·57	+7·63	<0·001*	−0·91	0·19	−0·04	0·95
B_12_, μg/d	7·47	−1·75	0·06	−1·49	0·10	−1·70	0·06	−2·02	0·03*	−1·88	0·04*	−1·76	0·05*

Fortification Model													
	Current	Fortified	*P*	Unfortified	*P*								
Energy, kJ/d	8995.60	–	–	–	–								
Carbohydrate, g/d	270·77	+11·24	0·003*	+23·26	<0·001*								
Protein, g/d	86·67	−3·09	0·005*	−18·19	<0·001*								
Fat, g/d	81·97	−4·90	<0·001*	−4·19	0·001*								
Fibre, g/d	21·86	+7·08	<0·001*	+8·37	<0·001*								
Sugars, g/d	110·52	+1·68	0·43	+4·12	0·06								
Saturated fat, g/d	30·96	−3·30	<0·001*	−3·94	<0·001*								
Na, mg/d	2413·80	+482·63	<0·001*	+361·09	<0·001*								
Fe, mg/d	13·65	+3·12	<0·001*	−0·54	0·43								
B_12_, μg/d	7·47	−0·33	0·72	−1·79	0·05*								

MA-25, replacement model substituting 25 % of self-reported meat intake with composite of meat alternatives; MA-50, replacement model substituting 50 % of self-reported meat intake with composite of meat alternatives; MA-75, replacement model substituting 75 % of self-reported meat intake with composite of meat alternatives; MA-100, replacement model substituting 100 % of self-reported meat intake with composite of meat alternatives.

Blue indicates significant increase compared with current. Gold indicates significant decrease compared with current.

*Differences compared using regression models with significance threshold *P* < 0.05.

#### Model 1: the effect of meat reduction and replacement with meat alternatives

In model 1, we explored projected changes in nutritional intake given a graded replacement of meat from replacing 25 % of current meat intake, through to a 100 % replacement, using weighted composite nutritional values for the meat alternatives based on consumer purchasing data. We observed a linear increase in total carbohydrate consumption with the decrease in meat, reaching an additional 19 g/d in a 100 % meat-replacement scenario. This increase in projected carbohydrates was statistically significant in all scenarios except the 25 % replacement model (MA-50, *P* = 0·018; MA-75, *P* ≤ 0·001; MA-100, *P* < 0·001). Importantly, the projected increased intake of carbohydrates was only associated with a small linear increase in projected sugar intake; this increase did not reach statistical significance until 100 % of current meat was replaced (+04·16 g/d, *P* = 0·05). In contrast, with decreasing consumption of meat and increasing consumption of meat alternatives, there was a graded decrease in projected total protein intake (MA-25, −3·40 g/d, *P* = 0·002; MA-50, −6·99 g/d, *P* ≤ 0·001; MA-75, −10·77 g/d, *P* ≤ 0·001; MA-100, −14·77 g/d, *P* < 0·001). There was also a linear decrease in the intake of fat and importantly, of saturated fat, with decreasing meat and increasing meat alternative intake. The decrease in saturated fat with complete meat substitution was equivalent to 3·96 g/d (*P* < 0·001).

Total fibre intake was increased significantly under each meat-replacement scenario. A 100 % replacement of meat with meat alternatives was associated with an 8·15 g increase in total daily fibre intake (*P* < 0·001). Notably, there was also a linear increase in Na intake, with a total replacement of meat being associated with a projected 228·03 mg increase in Na per day (*P* < 0·001). There were non-statistically significant decreases in projected intake of both Fe and of vitamin B_12_.

Similar effects were found in sub analyses across each of the population subgroups (Supplementary Tables S3–S18).

#### Model 2: a comparison of the effects of replacing nutrients from meat across specific categories of alternative

This model is based on a 100 % replacement of meat with the mean nutrient values from each of the separate categories of meat alternative. Compared with the current intake, the substitution of meat, with foods from any of the meat alternative categories, led to a meaningful increase in the projected mean intake of fibre. The greatest projected increase in total fibre was observed in models replacing meat with nut-based products (10·23 g/d), followed closely by substitution with vegetable (8·73 g/d) or mycoprotein-based products (8·71 g/d). Substitution with tofu-based products had the least impact on projected total fibre intake (+3·14 g/d).

All product categories were associated with meaningful reductions in projected saturated fat intake. The greatest reduction in saturated fat intake is projected for substitutions with nut-based meat alternatives (–4·8 g/d, *P* ≤ 0·001), whilst the greatest reduction in total projected fat intake was associated with substitution for mycoprotein (6·12 g/d, *P* ≤ 0·001). Only the replacements with nut-based and tofu-based meat alternatives did not cause significant reductions in projected total fat intake.

Conversely, projected intakes of protein were lower across each meat alternative category. The greatest projected reductions in total protein intake were observed when substituting in products exclusively from the nut (–19·79 g/d), vegetable (–19·33 g/d) or legume (–15·43 g/d) categories. Tofu had the least projected impact on total protein consumption, but it was still associated with a significant reduction in daily intake (–4·87 g/d). Replacement of meat with meat alternatives was also associated with an increase in total projected Na consumption except for the nut-based products (–124·30 mg/d). Soya-based meat alternatives had the highest Na content, and replacement with the soya product category raised projected Na intake by almost 0·5 g/d.

Regarding micronutrients, the projected intake of vitamin B_12_ was shown to be statistically significantly reduced in the replacement models with tofu (–2·02 μg/d; *P* = 0·03), nut (–1·88 μg/d; *P* = 0·04) and soya (–1·76 μg/d; *P* = 0·05), although in each case mean projected intake across the population remained in excess of the RNI.

Total Fe intake was projected to increase in the substitution model with tofu (+7·63 mg/d, *P* < 0·001). It was not significantly affected by the other meat-replacement scenarios.

In sub-analyses by population group (age and gender), the described influence of these substitution scenarios was replicated in each of the demographics (Supplementary Tables S3–S18).

#### Model 3: substitution with fortified *v*. unfortified meat alternatives

In this model, we compared the impact of selecting Fe and B_12_ fortified *v*. unfortified, meat alternatives, assuming a 100 % replacement of meat. Fortified products accounted for 14 % of the meat alternative products. The projected intake of Fe was significantly higher than current intakes when choosing fortified meat alternatives (+3·12 mg/d; *P* < 0·001) and would comfortably attain the UK dietary recommendations (fortified, 16·77 mg/d *v*. 14·8 mg/d RNI for girls and women aged 11–50, 8·7 mg/d RNI for remaining population). The projected intake of Fe was not statistically significantly reduced from current levels of intake in the unfortified model.

The projected intake of vitamin B_12_ was significantly decreased compared with current intakes in the unfortified model (–1·79 μg/d, *P* = 0·05); however, projected mean intake did not fall below the RNI. In the fortified model, projected intakes of vitamin B_12_ were not significantly different from current levels of intake.

Interestingly, fortified meat alternatives were associated with a much smaller projected reduction in total protein from current intakes (–3·09 g/d *v*. −18·19 g/d for the unfortified products *P* = 0·005); however, it was also associated with a greater projected increase in total Na from current levels of intake (–482 mg/d *v*. +361 mg/d for the unfortified products *P* < 0·001).

In sub analyses by population group (age and gender), the effect of choosing fortified products described above were replicated across subgroups (Supplementary Tables S3–S18).

## Discussion

We have modelled the projected nutritional impact of a shift in the culinary practices of the meat-eating UK population towards favouring meat alternative products and have identified some important nutritional benefits. Notably, our models predict significant increases in fibre intake and significant decreases in both total fat and saturated fat intake as people switch from meat to meat-alternatives. On the other hand, not all projected impacts on nutrient intake would be described as beneficial; there were notable reductions in total protein and vitamin B_12_ and projected increases in the intake of Na and of sugars.

The UK dietary guidelines recommend limiting fat to no more than 35 % of total energy. This target is generally met by the UK population, but further displacement of fat with fibre may have the potential to reduce total energy intake with consequences for body composition. We observed significant reductions in projected total fat intake across our model scenarios, with the exception of the nut and tofu-based alternatives. The greatest projected reduction in fat was associated with mycoprotein; if we assumed no dietary adjustment for total energy, daily replacement of meat with mycoprotein might be projected to reduce fat intake by the equivalent of 225.94 kJ/d, a level of reduction more than sufficient to offset weight gain^(39)^.

The UK guidelines set an upper limit of 11 % of total energy for saturated fat due to its association with CVD^(33,37)^. Actual intakes of saturated fat remain above this threshold despite long-running public health initiatives. We observed that a 100 % replacement of meat with meat alternatives would take total projected saturated fat intake very close to the 11 % of food energy target. This observation was consistent across the categories of meat alternative, but with the greatest projected reductions in saturated fat being observed with the nut-based and tofu-based products categories. Nut- and tofu-based meat alternatives may be of further interest as the projected displacement of saturated fat was with MUFA more so than carbohydrate^(40–42)^. Swapping SFA, for MUFA and PUFA, is considered optimal for heart health^(43)^.

The RNI for protein is greatly exceeded by most UK consumers pursuing an omnivorous, energy balanced diet. However, in our models, the projected reduction in total protein intake from a 100 % replacement of meat was equivalent to almost 20 g/d if alternative foods were drawn exclusively from the vegetable- or nut-based product categories. These losses have the potential to compromise protein status in athletes, in the very young, in the aged and in certain disease states where requirements may be above the RNI. Where protein intake is a concern, we would recommend choosing products from the higher protein, tofu-, soya- and mycoprotein-based meat-alternative categories.

Dietary patterns high in fibre are associated with improved satiety, reduced risk of obesity, improved metabolic health and reduced risk of diverticular disease, cancer, cardiovascular disease and hypertension^(44,45)^; the UK dietary guidelines recommend that adults consume upwards of 30 g of AOAC fibre per day^(33,37)^. Despite consistent public health campaigns over many years, dietary fibre consumption amongst the UK public remains significantly below this recommendation^(46)^. Using a refined NDNS cohort, incorporating meat eaters only, we noted that current mean fibre intake is 21·86 g/d. In our models, under every meat-replacement scenario, projected fibre intake was shown to increase significantly compared with current intake for the total population. Importantly, the models showing a 100 % replacement of meat with meat alternatives would increase the mean projected fibre intake, at a population level, to 30 g/d (Table 4).

We noted considerable differences in fibre content between, and within, our meat alternative product categories, with the highest projected increase in fibre intake being associated with vegetable, nut and mycoprotein-based products. Not all fibre elicits the same physiological benefits^(47)^, and therefore it is important to note that fibre composition, and functional activity, is better described for soya-based^(48)^ and mycoprotein-based^(49)^ meat alternatives than it is the for the nut, vegetable and tofu categories of product.

Non-milk extrinsic sugar intake in the UK remains well above current guidelines which suggest limiting intake to no higher than 5 % of total energy intake. In our meat eating NDNS cohort, sugar intake was closer to 20 % of total energy intake. A high intake of sugars may be associated with obesity and poor dental health outcomes^(50)^; therefore, any intervention that raises sugar consumption must be viewed as problematic. We noted particularly high sugar levels in the nut-based and vegetable-based product categories and in the 100 % meat displacement scenarios involving these two product categories, projected sugar intake rose to almost 22 % of energy intake.

Due to its associations with hypertension, kidney disease and some cancers, dietary Na reduction has been the goal of a multipronged and effective public health campaign over the past two decades^(51)^. Intakes in the UK have decreased by clinically meaningful levels in recent years; in the meat eating NDNS cohort used in this analysis, mean Na intake was reported as being very close to the current guidelines. Unfortunately, many of the meat alternative products were found to be high in Na, and in the 100 % meat-replacement model, total projected Na intake was increased by 0·23 g. As with the other nutrients, there was considerable variation in the Na content of foods from within and across alternative product categories; of note, soya-based meat alternatives were found to be particularly high in Na, and a scenario with 100 % meat displacement for soya-based products was projected to raise Na intake by almost 0·5 g/d. We caution that our displacement models cannot fully account for salt added at the table or in the preparation of meat dishes.

Fe intake amongst girls and women in the UK is marginal with the mean intakes for both girls aged 11 to 18 years, and women aged 19 to 64 years below the RNI (56 % and 76 % of the RNI respectively), and further, 49 % of girls aged 11 to 18 years and 25 % of women aged 19 to 64 years have Fe intakes below the lower RNI^(52)^. There is also evidence of both Fe-deficiency anaemia (as indicated by low haemoglobin levels) and low Fe stores (plasma ferritin) in 9 % of older girls, 5 % of adult women and 2 % of older women^(52)^. Replacing meat with meat alternative products did not significantly reduce projected total Fe intake; in fact, replacement with either tofu or with Fe-fortified meat alternatives was associated with a significant increase in Fe intake. Therefore, if meat alternatives are integrated into the dietary habits of the UK population, fortification of these foods with Fe could facilitate the widespread attainment of RNI.

In the UK population, vitamin B_12_ deficiency is rarely due to poor dietary intake, but it can be induced by a poorly managed vegan diet^(54)^, and thus B_12_ intake should be considered in any public health approaches in reducing animal product intake. In our model scenarios, we observed slight reductions in B_12_, approaching statistical significance with a total replacement of meat, and particularly evident when the replacement products were from the nut and tofu product categories. This reduction in projected B_12_ intake was largely offset in the model with fortified alternatives. In our models, the projected intakes of B_12_ following meat replacement remained significantly above the RNI, and therefore might be deemed of limited public health consequence. However, we caution that we did not remove milk, eggs, and other dairy products from our models, and we would expect further reductions in B_12_ under those scenarios. For individuals pursuing a vegan diet, it remains sensible to choose fortified products and to consider B_12_ supplementation.

To our knowledge, this is the first study to consider the projected impact of this important, relatively novel, food category at a population level in the UK. The strengths of this work are that we leverage the National Dietary Nutrition Survey data set; this is a very well developed and informative record of food habits in the UK. We have also captured a broad picture of the nutritional quality of meat alternative products from within the UK market in 2020.

The limitations of this study are that we are reliant upon self-reported dietary intake data from the NDNS. The 4-day food record collection tool used in the NDNS can change habitual diet, and the heavy respondent work load may precipitate socio-economic representation bias in the cohort. We have also made subjective decisions in approaching the analysis; most notably, we chose to keep the energy content of the substitution models constant by increasing the nutritional intake uniformly across the data set after the substitution. The energy adjustment approach is consisted of previous literature evaluating the impacts of dietary substitutions ^(55,56)^. However, whilst we assume that individuals will maintain energy balance in a meat-replacement scenario, we cannot be certain which foods they are likely to adjust their intake of, and this could affect the overall projected nutritional intake. We were further limited by the depth of nutritional data and relatively poor characterisation of many of the meat alternatives. Across the product categories, mycoprotein and soya-based products were generally well characterised, but there was a paucity of B vitamin and mineral data for many of the product categories, which has been noted previously ^(35)^. This meant that we were limited in the nutrients we could assess. Data pertaining to the nutritional profile of meat alternatives within the McCance and Widdowson data set are scarce, and there is a need for greater analytical work to be done to update and formulate databases for these foods. If meat alternatives are to be regularly consumed as part of the diet in place of meat, publication of a more comprehensive characterisation of their nutritional composition would be welcome.

There are implications arising from this work for consumers, producers and food policy makers (Table 6). For food consumers, it is clear there are many nutritious approaches to reducing meat intake using meat alternatives. We note the variable density of different nutrients across meat substitute categories as summarised in Table 7, none of these substitutes fully replicates the nutritional composition of meat. If choosing to use meat alternatives, we therefore recommend using a variety of products from across the meat substitute categories. We also recommend choosing products high in protein and fibre and low in saturated fat and sugar; where possible, we would advise choosing products that are good sources of Fe and vitamin B_12_. Producers of meat alternatives could better serve consumers with stronger nutritional labelling and by focussing on Na and sugar reduction. Producers might also consider voluntary fortification with Fe and B_12_. Food policy makers might consider regulation around fortification, marketing and labelling to better guide consumer choice.

**Table 6.** Implications and recommendations

i/Food consumers	Consumers choosing to reduce their meat intake should understand that meat alternative products are a culinary replacement for meat, which differ in their nutritional profile. With the exception of tofu and mycoprotein, we caution that few meat alternative products have been well evaluated for their physiological effects in the consumer. We would therefore strongly recommend selecting meat alternatives based on the level of nutritional characterisation and the availability of high-quality evidence about their healthfulness. It is also important to consider the wider balance of the diet to ensure that the intake of nutrients found in meat is not compromised when reducing the intake.
ii/Producers	Producers of meat alternatives should be encouraged to focus on Na reduction, deeper nutritional characterisation, and where feasible, investigations evidencing the likely health benefits of their products. Given their market, voluntary product fortification with Fe and B_12_ might also be considered.
iii/Policy makers	Meat alternatives are intended to displace a nutritionally dense component of the diet, better labelling and nutritional characterisation would be in the interest of the food consumer. Further, in 1960 policy makers in the UK responded to the displacement of butter and animal fats from the diet with legislation instructing the mandatory fortification of margarines with vitamins A and D, there may soon be a need for a discussion about fortification, and the regulation of nutritional quality and healthfulness of this category of food; this discussion would be better informed by appropriate dietary intervention studies.

**Table 7.** Nutritional comparisons across meat substitute product category

Ingredient model	Carbohydrate (g/d)	Protein (g/d)	Fat (g/d)	Fibre (g/d)	Sugars (g/d)	Saturated fat (g/d)	Na (mg/d)	Fe (mg/d)	B_12_ (μg/d)
Vegetable	**↑**	**↓**	**↓**	**↑**	**↑**	**↓**	**↑**	–	–
Mycoprotein	**↑**	**↓**	**↓**	**↑**	+	**↓**	**↑**	–	–
Legume	**↑**	**↓**	**↓**	**↑**	+	**↓**	**↑**	–	–
Tofu	**↑**	**↓**	–	**↑**	+	**↓**	**↑**	**↑**	**↓**
Nut	**↑**	**↓**	+	**↑**	**↑**	**↓**	**↓**	–	**↓**
Soya	**↑**	**↓**	**↓**	**↑**	+	**↓**	**↑**	–	**↓**

+, Non-significant increase compared with current intake; -,non-significant decrease from current intake. **↑**, significant increase compared with current intake; **↓**, significant decrease from current intake.

### Conclusion

Reducing meat consumption will be a focus of public health and ecological food policy for the foreseeable future. It is therefore anticipated that meat alternatives will play an increasingly prominent role in the UK food plate. Consumer confidence in the new and dynamic meat alternative market will be an important aspect in facilitating the level of dietary change required to protect planetary and consumer health. Going forward, periodic monitoring of the nutritional quality and healthfulness of meat alternatives will be necessary to ensure that these foods compare well on a nutritional playing field and that nutrient analytical databases are kept up to date with this information. This will then aid better quality research and the compilation of more accurate recommendations that relate to public health policy.

## Supplementary material

For supplementary material accompanying this paper visit https://doi.org/10.1017/S0007114521002750. click here to view supplementary material
