# Supplementary material for: The nutritional impact of replacing dietary meat with meat alternatives in the UK: a modelling analysis using nationally representative data
Source: Br J Nutr. 2021 Jul 21;127(11):1731–41. doi: 10.1017/S0007114521002750 (PMC9201833; doi:10.1017/S0007114521002750)
Supplement: Supplementary file 1 [file S0007114521002750sup001.docx]

**The nutritional impact of replacing dietary meat with meat alternatives in the UK: a modelling analysis using nationally representative data**

**Supplementary Material**

**Table 1: Meat alternative products included in modelling analysis**

**Table 2: Mean nutrient intake with 95% confidence intervals for current and replacement models; for total population**

**Tables 3-4: Population subgroup result tables; males aged 4-10 years**

**Tables 5-6: Population subgroup result tables; females aged 4-10 years**

**Tables 7-8: Population subgroup result tables; males aged 11-18 years**

**Tables 9-10: Population subgroup result tables; females aged 11-18 years**

**Tables 11-12: Population subgroup result tables; males aged 19-64 years**

**Tables 13-14: Population subgroup result tables; females aged 19-64 years**

**Tables 15-16: Population subgroup result tables; males aged 65 years and older**

**Tables 17-18: Population subgroup result tables; females aged 65 years and older**

| **Supplementary Table 1. Meat alternative products included in modelling analysis** | |
| --- | --- |
| **Soy based** | |
| **Brand/Manufacturer** | **Product Name** |
| Tesco | Meat Free Vegetarian Mince |
| Tesco | Meat Free Chicken Style Nuggets |
| Tesco | Meat Free Meat Style |
| Richmond | Frozen Meat Free Sausages |
| Richmond | Meat Free Sausages |
| Fry's Meat Free | Chicken Style Strips |
| Fry's Meat Free | Chicken Style Burger |
| Tesco | Vegetarian Bacon Mac & Cheese Melts |
| Aunt Bessie's | Vegetarian Toad In The Hole |
| Linda McCartney’s | Vegetarian Chicken Bucket |
| Naked Glory | Vegan Tikka Tenderstrips |
| Vivera | Veggie Greek Kebab∞ |
| Oumph! | Spiced Kebab |
| Vivera | Veggie Bacon Pieces∞ |
| Naked Glory | Vegan Quarter Pounders |
| Naked Glory | Vegan Sausages Pack |
| Oumph! | Sticky Smokehouse Oumph |
| Tesco | Plant Chef Herby Bangers |
| Tesco | Plant Chef Meat Free Balls |
| Linda McCartney’s | Vegetarian Red Onion & Rosemary Sausages |
| Linda McCartney’s | Vegetarian Quarter Pounder Burgers |
| Linda McCartney’s | Vegetarian Chorizo & Red Pepper Sausages |
| Like Meat | Soya Based Schnitzel |
| Naked Glory | Mince |
| Tesco | Plant Chef Breaded Goujons |
| Linda McCartney’s | Mozzarella Burgers |
| Tesco | Plant Chef Southern Fried Fillets |
| Tesco | Plant Chef Breaded Meat Free Nuggets |
| Vivera | Veggie Mince∞ |
| Naked Glory | Quarter Pounders Pack |
| The Vegetarian Butcher | What The Cluck |
| Naked Glory | Sausages Pack |
| Vivera | Plant Chicken Tenders∞ |
| Vivera | Veggie Chicken Pieces∞ |
| The Vegetarian Butcher | Chickened Out Burger∞ |
| The Unbelievable Alt. | Lambless Strips |
| Tesco | Plant Chef Fish Free Cakes |
| THIS | Isn't Chicken Salt & Pepper∞ |
| The Unbelievable Alt. | Beefless Strips |
| Fry's Meat Free | Golden Crispy Fish Style Fillets |
| Oumph! | Pulled Oumph! |
| The Vegetarian Butcher | Little Peckers (Nuggets)∞ |
| Vivera | Plant Meatballs∞ |
| The Vegetarian Butcher | Mc2 Burger∞ |
| The Vegetarian Butcher | Little Willies Sausages |
| Like Meat | Soya Based Nuggets |
| Linda McCartney’s | Shredded Hoisin Duck |
| Vivera | Plant Quarter Pounder∞ |
| The Unbelievable Alt. | Mighty Beefless Mince |
| Tesco | Plant Chef Battered Fish Free Fillets |
| Linda McCartney’s | BBQ Pulled Pork Burger |
| Wicked Kitchen | All The Rage Rolls |
| Fry's Meat Free | Chicken Style Sausage Rolls |
| Linda McCartney’s | Vegetarian Pork Bao Bun Meal Kit |
| THIS | Isn't Bacon Rashers∞ |
| Vivera | Shwarma Kebab∞ |
| The Unbelievable Alt. | Chickenless Strips |
| Vivera | Veggie Burger∞ |
| Sainsbury's | Plant Pioneers Meat Free Mince |
| Linda McCartney’s | Chilled Vegetarian Meatballs |
| Linda McCartney’s | Vegetarian Sausages |
| Fry's Meat Free | Vegan Meat Free Chicken-Style Nuggets |
| Sainsbury's | Plant Pioneers Meat Free Chicken-Style Pieces |
| Sainsbury's | Plant Pioneers Meat Free Mini Sausage Rolls |
| Linda McCartney’s | Vegetarian Fishcake |
| Sainsbury's | Plant Pioneers Ultimate Plant Burgers |
| Fry's Meat Free | Vegan Artisan Smoked Hot Dogs |
| Sainsbury's | Plant Pioneers Vegetarian Chicken Nuggets |
| Vivera | Plant Steak∞ |
| Gardein | Golden Fishless Fillets |
| Fry's Meat Free | Vegan Mini Sausage Rolls |
| Fry's Meat Free | Vegan Artisan Southern-Style Tenders |
| Gardein | Seven Grain Crispy Tenders |
| Gardein | Sweet and Sour Porkless Bites |
| Moving Mountains | Plant-Based Sausage Burgers |
| Fry's Meat Free | Vegan Twin Sausage Rolls |
| Squeaky | Satay Kiev |
| Squeaky | Vegan Nuggets |
| Asda | Plant Based Vegan Meat Free Chicken Bites |
| Asda | Plant Based Vegan Meat-Free Chicken Nuggets |
| Asda | Plant Based Vegan Meat Free Meatballs |
| Asda | Plant Based Vegan Meat Free Mince |
| Asda | Plant Based Vegan Quarter Pounders with Cheddar Alternative & Red Onion |
| Asda | Plant Based 8 Vegan Beef Style Burgers |
| Oumph! | Vegan Burger |
| No Chick Meat | Meat Free Strips |
| Linda McCartney’s | Vegetarian Southern-Style Chicken Fillet Burgers |
| **Nut Based** | |
| **Brand/Manufacturer** | **Product Name** |
| Tesco | Plant Chef Nut Cutlets |
| Tesco | Plant Chef Peanut Burgers |
| **Vegetable Based** | |
| **Brand/Manufacturer** | **Product Name** |
| Tesco | Vegetarian Cauliflower Cheese Grills |
| Tesco | Plant Chef Vegetable Fingers |
| Tesco | Plant Chef Vegetable Burgers |
| Birds Eye | Vegetable Fingers |
| Goodlife | Vegetable Protein Balls With Spinach & Kale |
| Heck | Ultimate Vegan Burgers |
| Birds Eye | Green Cuisine Mexican Fajita Style Mix |
| Strong Roots | Pumpkin Spinach Burger |
| Strong Roots | Kale & Quinoa Burger |
| Strong Roots | Spinach Bites |
| Goodlife | Mighty Non Meaty Sausages |
| Strong Roots | Broccoli & Purple Carrot Bites |
| Vivera | Pumpkin Sweet Potato Burgers |
| Sainsbury's | Sweet Potato, Quinoa & Lentil Burger |
| Sainsbury's | Vegetable Quarter Pounders |
| Sainsbury's | Indian Lentil Quarter Pounder |
| Sainsbury's | Love Your Veg! Vegan Mix Burger |
| Sainsbury's | Plant Pioneers Chorizo Shroomdogs |
| Sainsbury's | Plant Pioneers Smoky ‘Jack' Quarter Pounders |
| Sainsbury's | Plant Pioneers Cumberland Shroomdogs |
| Heck | Vegan Breakfast Plant Based Sausages |
| Sainsbury's | Plant Pioneers Cumberland Shroomdogs |
| Sainsbury's | Plant Pioneers Vegan Steaks∞ |
| Sainsbury's | Plant Pioneers Shroomballs |
| Sainsbury's | Plant Pioneers Hotnspicy Goujons |
| Sainsbury's | Love Your Veg! Green Supreme Shroomdogs Sausages |
| Sainsbury's | Plant Pioneers Caramelised Onion Shroomdogs |
| Sainsbury's | Love Your Veg! Roasted Red Pepper Shroomdogs |
| Sainsbury's | Plant Pioneers Fishless Fingers |
| Moving Mountains | Plant-Based Hot Dogs |
| Sainsbury's | Plant Pioneers Southern Fried Bites |
| Sainsbury's | Vegetable Fingers |
| Moving Mountains | Plant-Based Hot Dogs |
| Little Roots | Sweet Potato Nuggets |
| Sainsbury's | Moroccan Vegbab |
| Goodlife | Supergreen Pesto Burger with Edamame Beans, Kale & Spinach |
| Rustlers | Moroccan Vegetarian Burger |
| Heck | Vegfurter |
| Asda | Plant Based Meat-Free Mince |
| Asda | Plant Based Meat-Free Burgers |
| Asda | Plant Based Meat-Free Sausages |
| Asda | Plant Based Vegan Vegetable Burgers |
| Asda | Plant Based Vegan Vegetable Fingers |
| Asda | Plant Based Vegan Nut Cutlets |
| Asda | Plant Based Vegan Sweet Potato Falafel Burgers |
| Asda | Plant Based Meat-Free Meatballs |
| No Bull | Vegan Mushrooms Steaks |
| Asda | Vegetarian Cauliflower Cheese Crispy Grills |
| **Tofu Based** | |
| **Brand/Manufacturer** | **Product Name** |
| Cauldron | Organic Tofu |
| Cauldron | Marinated Tofu |
| Satono Yukis | Tofu |
| The Tofoo Co | Smoked Organic Tofu |
| Yutaka | Tofu |
| The Tofoo Co | Tempeh |
| Cauldron | Organic Teriyaki Tofu |
| The Tofoo Co | Organic Tofu |
| Clearspring Organic | Organic Tofu |
| Mori-Nu | Silken Firm Tofu |
| Plant Power | Original Tempeh Block |
| Tofurky | Hickory Smoke Flavour Turkey Style Deli Slices |
| Tofurky | Italian Sausage with Sun-Dried Tomatoes and Basil |
| The Tofoo Co | The Tofoo Co. Crispy Southern Fried Bites |
| Tofurky | Plant-Based Deli Slices Smoke Ham Style |
| Tofurky | Oven Roasted Deli Slices |
| The Tofoo Co | Crispy Spicy Sweet Chilli Bites |
| Satonoyuki | Tofu |
| Ashoka | Palak Paneer |
| Ashoka | Matar Paneer |
| **Mycoprotein Based** | |
| **Brand/Manufacturer** | **Product Name** |
| Quorn | Chicken Style Pieces |
| Quorn | Mince |
| Quorn | Crispy Nuggets |
| Quorn | Sausages |
| Quorn | Cheese Escalopes |
| Quorn | Southern Fried Bites |
| Quorn | Cheese & Broccoli Escalopes |
| Quorn | Chicken Style Burgers |
| Quorn | Love It Lasagne |
| Quorn | Tantalising Tikka Masala |
| Quorn | Family Roast |
| Quorn | Vegan Smoky Ham Free Slice |
| Quorn | Roast Style Sliced Fillets |
| Quorn | Chicken Style Pieces |
| Quorn | Brilliant Burgers |
| Quorn | Cocktail Sausages |
| Quorn | Crispy Fillets |
| Quorn | Comforting Cottage Pie |
| Quorn | Picnic Egg |
| Quorn | Pieces |
| Quorn | Sausage Rolls |
| Quorn | Mince |
| Quorn | Fishless Fillets Salt And Vinegar |
| Quorn | Bacon Style Rashers |
| Quorn | Chicken & Bacon Lattice |
| Quorn | Peppered Steaks |
| Quorn | Mince |
| Quorn | Classic Burger |
| Quorn | Vegan Chicken Free Slices |
| Quorn | Mozzarella & Pesto Escalope |
| Quorn | Vegetarian Ham Slices |
| Quorn | Ultimate Burgers |
| Quorn | Chilli Bean Bowl |
| Quorn | Smoky Bacon Style Slices |
| Quorn | Vegetarian Chicken Slices |
| Quorn | Biryani Bowl |
| Quorn | Hot & Spicy Vegan Burger |
| Quorn | Spiced Chickpea &Lentil Bowl |
| Quorn | Vegan Pieces |
| Quorn | Spinach and Red Pepper Slices |
| Quorn | Sweet Chipotle Goujons |
| Quorn | Thai Style Wonder Grains |
| Quorn | Mediterranean Wonder Grains |
| Quorn | Mexican 3 Bean Wonder Grain |
| Quorn | Ginsters Vegan Pasty |
| Quorn | Deli Turkey |
| **Legume Based** | |
| **Brand/Manufacturer** | **Product Name** |
| Birds Eye | Meat Free Swedish Style Balls |
| Birds Eye | Meat Free Burgers |
| Beyond Meat | Beyond Burger Plant Based Burger |
| Birds Eye | Green Cuisine Meat Free Chicken Pies |
| Birds Eye | Meat Free Sausages Rolls |
| Wicked Kitchen | Chorizo Style Bangers |
| The Meatless Farm | Meat Free Sausages |
| Beyond Meat | Plant Based Patties |
| Tesco | Plant Chef Cumberland Style Bangers |
| Tesco | Plant Chef Meat Free Burgers |
| Linda McCartney’s | Vegetarian Lincolnshire Sausages |
| The Meatless Farm | Meat Free Burgers |
| Heck | Meat-Free Vegan Sausages |
| Birds Eye | Green Cuisine Meat Free Bolognese Mix |
| Birds Eye | Green Cuisine Meat Free Chilli Mix |
| Tesco | Plant Chef Meat-Free BBQ Koftas |
| Wicked Kitchens | Chubby Little Brats |
| Wicked Kitchens | BBQ Fib Rack |
| Wicked Kitchens | Jalapeno Griller Patties |
| Like Meat | Pea Based Smoked Sausage |
| Beyond Meat | The Beyond Sausage Pack |
| Wicked Kitchens | Wicked Spiced Amazeballs |
| Squeaky | Ready To Eat Chicken Style Pieces Thai |
| Squeaky | Ready To Eat Chicken Style Pieces Tikka |
| Squeaky | Ready To Eat Chicken Style Pieces BBQ |
| Linda McCartney’s | Vegetarian Lincolnshire Sausages |
| Sainsbury's | Plant Pioneers Smoky Vacon Rashers∞ |
| Naturli | Pea Based Minced |
| Squeaky | Ready To Eat Marinated Chicken Style Pieces Kick of Tikka |
| Squeaky | Ready To Eat Marinated Chicken Style Pieces BBQ |
| Beanit | Pan-Fry Protein Pieces |
| Beanit | Pan-Fry Protein Mince |
| Tesco | Monterey Jack BBQ Bean Burger |
| Tesco | Plant Chef Spicy Bean Burgers |
| Strong Roots | Bean Beetroot Burger |
| Sainsbury's | Spicy Bean Burgers |
| Asda | Plant Based Vegan Spicy Bean Burgers |
| ∞ Product description indicates fortification with either iron/B12/both iron and B12 | |

| **Supplementary Table 2.  Mean nutrient intake with 95% confidence intervals for total population (n = 1110) for current and replacement models** | | | | | | | | | | |
| --- | --- | --- | --- | --- | --- | --- | --- | --- | --- | --- |
| **Models** | **Energy, kcal/day** | **Carbohydrate, g/day** | **Protein, g/day** | **Fat,**  **g/day** | **Fibre, g/day** | **Sugars,**  **g/day** | **Saturated fat, g/day** | **Sodium,**  **mg/day** | **Iron, mg/day** | **B12, ug/day** |
| **Current** | 2151 (2111.63, 2190.36) | 270.77 (265.61, 275.92) | 86.67 (84.96, 88.39) | 81.97 (80.14, 83.80) | 21.86 (21.35, 22.38) | 110.52 (107.55, 113.49) | 30.96 (30.18, 31.74) | 2413.80 (2359.39, 2468.20) | 13.65 (12.70, 14.60) | 7.47 (6.20, 8.74) |
| **MA-25** | 2151 (2111.63, 2190.36) | 275.16 (269.96, 280.35) | 83.27 (81.63, 84.91) | 81.19 (79.37, 83.01) | 23.74 (23.21, 24.27) | 111.48 (108.50, 114.45) | 30.05 (29.27, 30.82) | 2466.37 (2411.03, 2521.70) | 13.59 (12.63, 14.54) | 7.07 (5.80, 8.34) |
| **MA-50** | 2151 (2111.63, 2190.36) | 279.78 (274.54, 285.01) | 79.68 (78.11, 81.25) | 80.38 (78.57, 82.18) | 25.71 (25.16, 26.26) | 112.49 (109.51, 115.47) | 29.08 (28.32, 29.85) | 2521.74 (2465.38, 2578.10) | 13.52 (12.57, 14.47) | 6.65 (5.38, 7.92) |
| **MA-75** | 2151 (2111.63, 2190.36) | 284.65 (279.37, 289.94) | 75.90 (74.40, 77.40) | 79.51 (77.71, 81.31) | 27.80 (27.22, 28.37) | 113.56 (110.57, 116.54) | 28.07 (27.31, 28.83) | 2580.14 (2522.66, 2637.61) | 13.45 (12.50, 14.40) | 6.21 (4.94, 7.48) |
| **MA-100** | 2151 (2111.63, 2190.36) | 289.80 (284.46, 295.15) | 71.90 (70.47, 73.33) | 78.60 (76.81, 80.39) | 30.00 (29.38, 30.61) | 114.68 (111.69, 117.68) | 27.00 (26.24, 27.75) | 2641.82 (2583.13, 2700.52) | 13.38 (12.43, 14.33) | 5.74 (4.48, 7.01) |
| **Vegetable** | 2151 (2111.63, 2190.36) | 295.39 (289.98, 300.81) | 67.34 (65.98, 68.70) | 77.58 (75.81, 79.36) | 30.59 (29.96, 31.22) | 114.94 (111.95, 117.94) | 27.13 (26.38, 27.89) | 2720.90 (2660.58, 2781.22) | 13.17 (12.22, 14.12) | 5.72 (4.46, 6.99) |
| **Mycoprotein** | 2151 (2111.63, 2190.36) | 293.17 (287.79, 298.56) | 77.86 (76.32, 79.39) | 75.85 (74.09, 77.60) | 30.57 (29.94, 31.20) | 113.30 (110.31, 116.28) | 27.31 (26.55, 28.06) | 2726.12 (2665.69, 2786.55) | 12.68 (11.73, 13.63) | 5.98 (4.72, 7.25) |
| **Legume** | 2151 (2111.63, 2190.36) | 290.68 (285.33, 296.04) | 71.24 (69.82, 72.66) | 78.48 (76.69, 80.27) | 28.66 (28.06, 29.25) | 113.93 (110.95, 116.92) | 27.23 (26.48, 27.99) | 2690.65 (2630.96, 2750.34) | 13.26 (12.31, 14.21) | 5.77 (4.50, 7.03) |
| **Tofu** | 2151 (2111.63, 2190.36) | 279.77 (274.53, 285.00) | 81.80 (80.19, 83.41) | 79.60 (77.81, 81.40) | 25.00 (24.46, 25.54) | 112.66 (109.68, 115.65) | 26.63 (25.88, 27.39) | 2510.20 (2454.05, 2566.34) | 21.28 (20.29, 22.28) | 5.45 (4.18, 6.71) |
| **Nut** | 2151 (2111.63, 2190.36) | 282.24 (276.98, 287.50) | 66.88 (65.53, 68.24) | 83.35 (81.50, 85.20) | 32.10 (31.44, 32.75) | 117.96 (114.94, 120.98) | 26.16 (25.41, 26.91) | 2289.49 (2237.15, 2341.84) | 12.74 (11.79, 13.69) | 5.59 (4.32, 6.85) |
| **Soy** | 2151 (2111.63, 2190.36) | 285.33 (280.03, 290.62) | 79.48 (77.91, 81.05) | 77.26 (75.49, 79.04) | 28.12 (27.53, 28.70) | 112.71 (109.73, 115.70) | 27.27 (26.52, 28.03) | 2909.77 (2845.33, 2974.21) | 13.61 (12.66, 14.56) | 5.71 (4.44, 6.97) |
| **Fortified** | 2151 (2111.63, 2190.36) | 282.01 (276.75, 287.26) | 83.58 (81.93, 85.23) | 77.08 (75.31, 78.85) | 28.94 (28.35, 29.54) | 112.20 (109.22, 115.18) | 27.66 (26.90, 28.42) | 2896.42 (2832.28, 2960.56) | 16.77 (15.80, 17.73) | 7.14 (5.87, 8.41) |
| **Unfortified** | 2151 (2111.63, 2190.36) | 294.03 (288.63, 299.42) | 68.49 (67.11, 69.86) | 77.78 (76.01, 79.56) | 30.23 (29.61, 30.85) | 114.63 (111.64, 117.63) | 27.02 (26.27, 27.78) | 2774.88 (2713.42, 2836.35) | 13.10 (12.15, 14.06) | 5.68 (4.42, 6.95) |
| Data presented as means with 95% confidence intervals within parentheses | | | | | | | | | | |

| **Supplementary Table 3.**  **Mean nutrient intake for current and replacement models, with reference to DRVs; males aged 4-10 years (n = 141)** | | | | | | | | | | | | | | |
| --- | --- | --- | --- | --- | --- | --- | --- | --- | --- | --- | --- | --- | --- | --- |
|  |  |  | **Progressive Model** | | | | **Ingredient Model** | | | | | | **Fortification Model** | |
| **Nutrient** | **DRV*** | **Current** | **MA-25** | **MA-50** | **MA-75** | **MA-100** | **Vegetable** | **Mycoprotein** | **Legume** | **Tofu** | **Nut** | **Soy** | **Fortified** | **Unfortified** |
| Energy, kcal/day | 1673 | - | - | - | - | - | - | - | - | - | - | - | - | - |
| Carbohydrate, g/day† | 209.10 | 229.12 | 232.64 | 236.34 | 240.25 | 244.38 | 248.86 | 247.08 | 245.09 | 236.34 | 238.32 | 240.79 | 238.13 | 247.76 |
| Protein,  g/day† | 62.70 | 62.13 | 59.40 | 56.53 | 53.50 | 50.29 | 46.64 | 55.07 | 49.76 | 58.23 | 46.27 | 56.37 | 59.65 | 47.55 |
| Fat,  g/day | 65.10 | 62.81 | 62.19 | 61.53 | 60.84 | 60.11 | 59.30 | 57.90 | 60.01 | 60.92 | 63.92 | 59.04 | 58.89 | 59.46 |
| Fibre,  g/day | 20.00 | 16.08 | 17.58 | 19.16 | 20.83 | 22.60 | 23.07 | 23.06 | 21.52 | 18.59 | 24.28 | 21.09 | 21.75 | 22.78 |
| Sugars,  g/day | 90.00 | 95.11 | 95.88 | 96.69 | 97.55 | 98.45 | 98.66 | 97.34 | 97.85 | 96.83 | 101.08 | 96.87 | 96.46 | 98.41 |
| Saturated fat, g/day | 20.50 | 24.48 | 23.75 | 22.98 | 22.16 | 21.30 | 21.41 | 21.55 | 21.49 | 21.01 | 20.63 | 21.52 | 21.83 | 21.32 |
| Sodium, mg/day | 1600 | 1852.01 | 1894.15 | 1938.53 | 1985.34 | 2034.78 | 2098.17 | 2102.38 | 2073.93 | 1929.28 | 1752.37 | 2249.58 | 2238.89 | 2141.44 |
| Iron,  mg/day | 7.40 | 9.35 | 9.30 | 9.24 | 9.19 | 9.13 | 8.96 | 8.57 | 9.03 | 15.47 | 8.62 | 9.31 | 11.84 | 8.91 |
| B12,  ug/day | 1 | 4.65 | 4.33 | 3.99 | 3.64 | 3.26 | 3.24 | 3.46 | 3.28 | 3.03 | 3.14 | 3.23 | 4.38 | 3.21 |
| *DRV values correspond to EAR for energy; RNI for Protein, Sodium, Iron, B12; DRV for Carbohydrate, Fat, Fibre, Sugars, Saturated fat.  † Protein target elected as 15% total energy intake and carbohydrate as 50% total energy intake in the current analysis.  Colours: Green, meeting DRV. Red, not meeting DRV. Grey, not meeting 15% protein and/or 50% carbohydrate target elected in the current analysis.  DRV, Dietary Reference Value; EAR, Estimated Average Requirement; RNI, Reference Nutrient Intake; MA-25, Replacement model substituting 25% of self-reported meat intake with composite of meat alternatives; MA-50, Replacement model substituting 50% of self-reported meat intake with composite of meat alternatives; MA-75, Replacement model substituting 75% of self-reported meat intake with composite of meat alternatives; MA-100, Replacement model substituting 100% of self-reported meat intake with composite of meat alternatives. | | | | | | | | | | | | | | |

| **Supplementary Table 4.  Difference in nutrient intake between replacement models and current intake; males aged 4-10 years (n = 141)** | | | | | | | | | | | | | | | | | | | | | | | | | |
| --- | --- | --- | --- | --- | --- | --- | --- | --- | --- | --- | --- | --- | --- | --- | --- | --- | --- | --- | --- | --- | --- | --- | --- | --- | --- |
|  |  | **Progressive Model** | | | | | | | | **Ingredient Model** | | | | | | | | | | | | **Fortification Model** | | | |
| **Nutrient** | **Current** | **MA-25** | **P** | **MA-50** | **P** | **MA-75** | **P** | **MA-100** | **P** | **Vegetable** | **P** | **Mycoprotein** | **P** | **Legume** | **P** | **Tofu** | **P** | **Nut** | **P** | **Soy** | **P** | **Fortified** | **P** | **Unfortified** | **P** |
| Energy, kcal/day | 1673 | - | - | - | - | - | - | - | - |  |  | - | - | - | - |  |  |  |  | - | - | - | - | - | - |
| Carbohydrate, g/day | 229.12 | +3.52 | 0.62 | +7.22 | 0.31 | +11.13 | 0.12 | +15.26 | 0.03* | +19.74 | 0.01* | +17.96 | 0.01* | +15.97 | 0.03* | +7.22 | 0.31 | +9.20 | 0.20 | +11.67 | 0.10 | +9.01 | 0.21 | +18.64 | 0.01* |
| Protein, g/day | 62.13 | -2.73 | 0.14 | -5.60 | <0.01* | -8.64 | <0.01* | -11.84 | <0.01* | -15.50 | <0.01* | -7.07 | <0.01* | -12.37 | <0.01* | -3.90 | 0.03* | -15.86 | <0.01* | -5.77 | <0.01* | -2.48 | 0.18 | -14.58 | <0.01* |
| Fat, g/day | 62.81 | -0.62 | 0.80 | -1.28 | 0.60 | -1.97 | 0.42 | -2.70 | 0.27 | -3.52 | 0.15 | -4.91 | 0.05* | -2.80 | 0.25 | -1.90 | 0.44 | +1.10 | 0.65 | -3.77 | 0.12 | -3.92 | 0.11 | -3.36 | 0.17 |
| Fibre, g/day | 16.08 | +1.50 | 0.04* | +3.09 | <0.01* | +4.76 | <0.01* | +6.52 | <0.01* | +6.99 | <0.01* | +6.98 | <0.01* | +5.44 | <0.01* | +2.52 | <0.01* | +8.20 | <0.01* | +5.01 | <0.01* | +5.68 | <0.01* | +6.71 | <0.01* |
| Sugars, g/day | 95.11 | +0.77 | 0.84 | +1.58 | 0.67 | +2.43 | 0.51 | +3.34 | 0.37 | +3.55 | 0.34 | +2.23 | 0.55 | +2.74 | 0.46 | +1.72 | 0.64 | +5.96 | 0.11 | +1.76 | 0.63 | +1.35 | 0.71 | +3.30 | 0.37 |
| Saturated fat, g/day | 24.48 | -0.73 | 0.51 | -1.50 | 0.17 | -2.32 | 0.04* | -3.18 | <0.01* | -3.07 | 0.01* | -2.93 | 0.01* | -2.99 | 0.01* | -3.47 | <0.01* | -3.85 | <0.01* | -2.96 | 0.01* | -2.65 | 0.02* | -3.16 | <0.01* |
| Sodium, mg/day | 1852.01 | +42.14 | 0.57 | +86.52 | 0.25 | +133.33 | 0.08 | +182.78 | 0.01* | +246.16 | <0.01* | +250.37 | <0.01* | +221.93 | <0.01* | +77.27 | 0.30 | -99.64 | 0.18 | +397.57 | <0.01* | +386.88 | <0.01* | +289.43 | <0.01* |
| Iron, mg/day | 9.35 | -0.05 | 0.89 | -0.10 | 0.78 | -0.16 | 0.67 | -0.22 | 0.56 | -0.38 | 0.30 | -0.78 | 0.04* | -0.31 | 0.40 | +6.12 | <0.01* | -0.73 | 0.05* | -0.03 | 0.93 | +2.50 | <0.01* | -0.44 | 0.24 |
| B12, ug/day | 4.65 | -0.32 | 0.14 | -0.66 | <0.01* | -1.01 | <0.01* | -1.39 | <0.01* | -1.40 | <0.01* | -1.19 | <0.01* | -1.37 | <0.01* | -1.62 | <0.01* | -1.51 | <0.01* | -1.41 | <0.01* | -0.27 | 0.22 | -1.43 | <0.01* |
| **Blue indicates significant increase compared to Current. Gold indicates significant decrease compared to Current.** Differences compared using regression models with significance threshold P < 0.05  MA-25, Replacement model substituting 25% of self-reported meat intake with composite of meat alternatives; MA-50, Replacement model substituting 50% of self-reported meat intake with composite of meat alternatives; MA-75, Replacement model substituting 75% of self-reported meat intake with composite of meat alternatives; MA-100, Replacement model substituting 100% of self-reported meat intake with composite of meat alternatives. | | | | | | | | | | | | | | | | | | | | | | | | | |

| **Supplementary Table 5.**  **Mean nutrient intake for current and replacement models, with reference to DRVs; females aged 4-10 years (n = 123)** | | | | | | | | | | | | | | |
| --- | --- | --- | --- | --- | --- | --- | --- | --- | --- | --- | --- | --- | --- | --- |
|  |  |  | **Progressive Model** | | | | **Ingredient Model** | | | | | | **Fortification Model** | |
| **Nutrient** | **DRV*** | **Current** | **MA-25** | **MA-50** | **MA-75** | **MA-100** | **Vegetable** | **Mycoprotein** | **Legume** | **Tofu** | **Nut** | **Soy** | **Fortified** | **Unfortified** |
| Energy, kcal/day | 1564 | - | - | - | - | - | - | - | - | - | - | - | - | - |
| Carbohydrate, g/day† | 195.50 | 212.90 | 216.17 | 219.62 | 223.27 | 227.11 | 231.28 | 229.63 | 227.77 | 219.62 | 221.47 | 223.77 | 221.29 | 230.26 |
| Protein,  g/day† | 58.60 | 57.89 | 55.34 | 52.67 | 49.84 | 46.86 | 43.45 | 51.30 | 46.36 | 54.25 | 43.11 | 52.52 | 55.58 | 44.31 |
| Fat,  g/day | 60.80 | 59.41 | 58.83 | 58.22 | 57.57 | 56.89 | 56.13 | 54.84 | 56.80 | 57.64 | 60.44 | 55.90 | 55.76 | 56.28 |
| Fibre,  g/day | 20.00 | 14.98 | 16.38 | 17.85 | 19.41 | 21.05 | 21.49 | 21.48 | 20.05 | 17.32 | 22.62 | 19.65 | 20.26 | 21.22 |
| Sugars,  g/day | 90.00 | 92.86 | 93.57 | 94.33 | 95.13 | 95.97 | 96.16 | 94.93 | 95.41 | 94.46 | 98.41 | 94.50 | 94.11 | 95.93 |
| Saturated fat, g/day | 19.10 | 23.39 | 22.71 | 21.99 | 21.23 | 20.43 | 20.53 | 20.66 | 20.60 | 20.16 | 19.80 | 20.63 | 20.92 | 20.45 |
| Sodium, mg/day | 1600 | 1685.42 | 1724.67 | 1766.01 | 1809.63 | 1855.68 | 1914.71 | 1918.63 | 1892.14 | 1757.40 | 1592.60 | 2055.74 | 2045.81 | 1955.04 |
| Iron,  mg/day | 7.40 | 8.44 | 8.39 | 8.35 | 8.29 | 8.24 | 8.08 | 7.72 | 8.15 | 14.14 | 7.76 | 8.41 | 10.77 | 8.04 |
| B12,  ug/day | 1 | 4.41 | 4.11 | 3.80 | 3.47 | 3.12 | 3.10 | 3.30 | 3.14 | 2.90 | 3.01 | 3.09 | 4.16 | 3.08 |
| *DRV values correspond to EAR for energy; RNI for Protein, Sodium, Iron, B12; DRV for Carbohydrate, Fat, Fibre, Sugars, Saturated fat.  † Protein target elected as 15% total energy intake and carbohydrate as 50% total energy intake in the current analysis.  Colours: Green, meeting DRV. Red, not meeting DRV. Grey, not meeting 15% protein and/or 50% carbohydrate target elected in the current analysis.  DRV, Dietary Reference Value; EAR, Estimated Average Requirement; RNI, Reference Nutrient Intake; MA-25, Replacement model substituting 25% of self-reported meat intake with composite of meat alternatives; MA-50, Replacement model substituting 50% of self-reported meat intake with composite of meat alternatives; MA-75, Replacement model substituting 75% of self-reported meat intake with composite of meat alternatives; MA-100, Replacement model substituting 100% of self-reported meat intake with composite of meat alternatives. | | | | | | | | | | | | | | |

| **Supplementary Table 6.  Difference in nutrient intake between replacement models and current intake; females aged 4-10 years (n = 123)** | | | | | | | | | | | | | | | | | | | | | | | | | |
| --- | --- | --- | --- | --- | --- | --- | --- | --- | --- | --- | --- | --- | --- | --- | --- | --- | --- | --- | --- | --- | --- | --- | --- | --- | --- |
|  |  | **Progressive Model** | | | | | | | | **Ingredient Model** | | | | | | | | | | | | **Fortification Model** | | | |
| **Nutrient** | **Current** | **MA-25** | **P** | **MA-50** | **P** | **MA-75** | **P** | **MA-100** | **P** | **Vegetable** | **P** | **Mycoprotein** | **P** | **Legume** | **P** | **Tofu** | **P** | **Nut** | **P** | **Soy** | **P** | **Fortified** | **P** | **Unfortified** | **P** |
| Energy, kcal/day | 1564.00 | - | - | - | - | - | - | - | - |  |  | - | - | - | - |  |  |  |  | - | - | - | - | - | - |
| Carbohydrate, g/day | 212.90 | +3.28 | 0.61 | +6.73 | 0.29 | +10.37 | 0.11 | +14.22 | 0.03* | +18.39 | <0.01* | +16.73 | 0.01* | +14.87 | 0.02* | +6.72 | 0.29 | +8.57 | 0.18 | +10.87 | 0.09 | +8.39 | 0.19 | +17.37 | 0.01* |
| Protein, g/day | 57.89 | -2.54 | 0.11 | -5.22 | <0.01* | -8.04 | <0.01* | -11.03 | <0.01* | -14.44 | <0.01* | -6.58 | <0.01* | -11.52 | <0.01* | -3.64 | 0.02* | -14.78 | <0.01* | -5.37 | <0.01* | -2.31 | 0.15 | -13.58 | <0.01* |
| Fat, g/day | 59.41 | -0.58 | 0.79 | -1.19 | 0.58 | -1.84 | 0.39 | -2.52 | 0.24 | -3.28 | 0.13 | -4.57 | 0.03* | -2.61 | 0.23 | -1.77 | 0.41 | +1.03 | 0.63 | -3.51 | 0.10 | -3.66 | 0.09 | -3.13 | 0.15 |
| Fibre, g/day | 14.98 | +1.40 | 0.02* | +2.87 | <0.01* | +4.43 | <0.01* | +6.07 | <0.01* | +6.52 | <0.01* | +6.50 | <0.01* | +5.07 | <0.01* | +2.34 | <0.01* | +7.64 | <0.01* | +4.67 | <0.01* | +5.29 | <0.01* | +6.25 | <0.01* |
| Sugars, g/day | 92.86 | +0.72 | 0.85 | +1.47 | 0.70 | +2.27 | 0.56 | +3.11 | 0.42 | +3.31 | 0.39 | +2.07 | 0.59 | +2.55 | 0.51 | +1.60 | 0.68 | +5.56 | 0.15 | +1.64 | 0.67 | +1.26 | 0.74 | +3.07 | 0.42 |
| Saturated fat, g/day | 23.39 | -0.68 | 0.45 | -1.40 | 0.12 | -2.16 | 0.02* | -2.96 | <0.01* | -2.86 | <0.01* | -2.73 | <0.01* | -2.78 | <0.01* | -3.23 | <0.01* | -3.58 | <0.01* | -2.75 | <0.01* | -2.46 | 0.01* | -2.94 | <0.01* |
| Sodium, mg/day | 1685.42 | +39.25 | 0.59 | +80.59 | 0.26 | +124.21 | 0.09 | +170.26 | 0.02* | +229.29 | <0.01* | +233.21 | <0.01* | +206.72 | <0.01* | +71.99 | 0.32 | -92.82 | 0.20 | +370.32 | <0.01* | +360.39 | <0.01* | +269.62 | <0.01* |
| Iron, mg/day | 8.44 | -0.05 | 0.89 | -0.10 | 0.78 | -0.15 | 0.66 | -0.20 | 0.55 | -0.36 | 0.29 | -0.72 | 0.03 | -0.29 | 0.39 | +5.70 | <0.01* | -0.68 | 0.05* | -0.03 | 0.93 | +2.33 | <0.01* | -0.41 | 0.23 |
| B12, ug/day | 4.41 | -0.30 | 0.18 | -0.61 | 0.01* | -0.94 | <0.01* | -1.29 | <0.01* | -1.31 | <0.01* | -1.11 | <0.01* | -1.27 | <0.01* | -1.51 | <0.01* | -1.41 | <0.01* | -1.32 | <0.01* | -0.25 | 0.26 | -1.34 | <0.01* |
| **Blue indicates significant increase compared to Current. Gold indicates significant decrease compared to Current.** Differences compared using regression models with significance threshold P < 0.05  MA-25, Replacement model substituting 25% of self-reported meat intake with composite of meat alternatives; MA-50, Replacement model substituting 50% of self-reported meat intake with composite of meat alternatives; MA-75, Replacement model substituting 75% of self-reported meat intake with composite of meat alternatives; MA-100, Replacement model substituting 100% of self-reported meat intake with composite of meat alternatives. | | | | | | | | | | | | | | | | | | | | | | | | | |

| **Supplementary Table 7.**  **Mean nutrient intake for current and replacement models, with reference to DRVs; males aged 11-18 years (n = 112)** | | | | | | | | | | | | | | |
| --- | --- | --- | --- | --- | --- | --- | --- | --- | --- | --- | --- | --- | --- | --- |
|  |  |  | **Progressive Model** | | | | **Ingredient Model** | | | | | | **Fortification Model** | |
| **Nutrient** | **DRV*** | **Current** | **MA-25** | **MA-50** | **MA-75** | **MA-100** | **Vegetable** | **Mycoprotein** | **Legume** | **Tofu** | **Nut** | **Soy** | **Fortified** | **Unfortified** |
| Energy, kcal/day | 2680 | - | - | - | - | - | - | - | - | - | - | - | - | - |
| Carbohydrate, g/day† | 335.00 | 359.13 | 364.00 | 369.13 | 374.54 | 380.25 | 386.45 | 383.98 | 381.23 | 369.12 | 371.86 | 375.28 | 371.60 | 384.93 |
| Protein,  g/day† | 100.50 | 104.21 | 100.44 | 96.46 | 92.26 | 87.83 | 82.77 | 94.43 | 87.09 | 98.81 | 82.26 | 96.23 | 100.78 | 84.04 |
| Fat,  g/day | 104.20 | 100.80 | 99.94 | 99.03 | 98.07 | 97.06 | 95.94 | 94.01 | 96.93 | 98.18 | 102.33 | 95.58 | 95.37 | 96.16 |
| Fibre,  g/day | 30.00 | 24.05 | 26.13 | 28.33 | 30.64 | 33.08 | 33.74 | 33.71 | 31.59 | 27.54 | 35.41 | 30.99 | 31.91 | 33.34 |
| Sugars,  g/day | 90.00 | 137.19 | 138.26 | 139.38 | 140.56 | 141.81 | 142.10 | 140.27 | 140.98 | 139.57 | 145.45 | 139.63 | 139.06 | 141.76 |
| Saturated fat, g/day | 32.80 | 38.69 | 37.68 | 36.61 | 35.48 | 34.30 | 34.44 | 34.64 | 34.56 | 33.89 | 33.37 | 34.60 | 35.03 | 34.32 |
| Sodium, mg/day | 2400 | 2969.08 | 3027.40 | 3088.82 | 3153.61 | 3222.03 | 3309.76 | 3315.57 | 3276.21 | 3076.02 | 2831.19 | 3519.28 | 3504.49 | 3369.66 |
| Iron,  mg/day | 8.70 | 14.42 | 14.35 | 14.28 | 14.20 | 14.12 | 13.89 | 13.35 | 13.99 | 22.89 | 13.42 | 14.38 | 17.88 | 13.82 |
| B12,  ug/day | 1.5 | 6.93 | 6.49 | 6.02 | 5.53 | 5.01 | 4.99 | 5.28 | 5.04 | 4.69 | 4.84 | 4.97 | 6.56 | 4.94 |
| *DRV values correspond to EAR for energy; RNI for Protein, Sodium, Iron, B12; DRV for Carbohydrate, Fat, Fibre, Sugars, Saturated fat.  † Protein target elected as 15% total energy intake and carbohydrate as 50% total energy intake in the current analysis.  Colours: Green, meeting DRV. Red, not meeting DRV. Grey, not meeting 15% protein and/or 50% carbohydrate target elected in the current analysis.  DRV, Dietary Reference Value; EAR, Estimated Average Requirement; RNI, Reference Nutrient Intake; MA-25, Replacement model substituting 25% of self-reported meat intake with composite of meat alternatives; MA-50, Replacement model substituting 50% of self-reported meat intake with composite of meat alternatives; MA-75, Replacement model substituting 75% of self-reported meat intake with composite of meat alternatives; MA-100, Replacement model substituting 100% of self-reported meat intake with composite of meat alternatives. | | | | | | | | | | | | | | |

| **Supplementary Table 8.  Difference in nutrient intake between replacement models and current intake; males aged 11-18 years (n = 112)** | | | | | | | | | | | | | | | | | | | | | | | | | |
| --- | --- | --- | --- | --- | --- | --- | --- | --- | --- | --- | --- | --- | --- | --- | --- | --- | --- | --- | --- | --- | --- | --- | --- | --- | --- |
|  |  | **Progressive Model** | | | | | | | | **Ingredient Model** | | | | | | | | | | | | **Fortification Model** | | | |
| **Nutrient** | **Current** | **MA-25** | **P** | **MA-50** | **P** | **MA-75** | **P** | **MA-100** | **P** | **Vegetable** | **P** | **Mycoprotein** | **P** | **Legume** | **P** | **Tofu** | **P** | **Nut** | **P** | **Soy** | **P** | **Fortified** | **P** | **Unfortified** | **P** |
| Energy, kcal/day | 2680.00 | - | - | - | - | - | - | - | - |  |  | - | - | - | - |  |  |  |  | - | - | - | - | - | - |
| Carbohydrate, g/day | 359.13 | +4.87 | 0.69 | +10.00 | 0.42 | +15.41 | 0.21 | +21.12 | 0.09 | +27.32 | 0.03* | +24.85 | 0.04* | +22.10 | 0.07 | +9.99 | 0.42 | +12.73 | 0.30 | +16.15 | 0.19 | +12.47 | 0.31 | +25.80 | 0.04* |
| Protein, g/day | 104.21 | -3.78 | 0.34 | -7.76 | 0.05* | -11.95 | <0.01* | -16.38 | <0.01* | -21.45 | <0.01* | -9.78 | 0.01* | -17.12 | <0.01* | -5.40 | 0.17 | -21.95 | <0.01* | -7.98 | 0.04* | -3.43 | 0.39 | -20.18 | <0.01* |
| Fat, g/day | 100.80 | -0.86 | 0.84 | -1.77 | 0.68 | -2.73 | 0.53 | -3.74 | 0.39 | -4.87 | 0.26 | -6.79 | 0.12 | -3.87 | 0.37 | -2.62 | 0.54 | +1.53 | 0.72 | -5.22 | 0.23 | -5.43 | 0.21 | -4.64 | 0.28 |
| Fibre, g/day | 24.05 | +2.08 | 0.07 | +4.27 | <0.01* | +6.58 | <0.01* | +9.02 | <0.01* | +9.68 | <0.01* | +9.66 | <0.01* | +7.54 | <0.01* | +3.48 | <0.01* | +11.35 | <0.01* | +6.94 | <0.01* | +7.86 | <0.01* | +9.28 | <0.01* |
| Sugars, g/day | 137.19 | +1.06 | 0.89 | +2.19 | 0.78 | +3.37 | 0.67 | +4.62 | 0.56 | +4.91 | 0.53 | +3.08 | 0.69 | +3.79 | 0.63 | +2.38 | 0.76 | +8.25 | 0.29 | +2.44 | 0.76 | +1.87 | 0.81 | +4.57 | 0.56 |
| Saturated fat, g/day | 38.69 | -1.01 | 0.63 | -2.08 | 0.32 | -3.21 | 0.12 | -4.40 | 0.04* | -4.25 | 0.04* | -4.05 | 0.05* | -4.14 | 0.05* | -4.80 | 0.02* | -5.32 | 0.01* | -4.09 | 0.05* | -3.66 | 0.08 | -4.37 | 0.04* |
| Sodium, mg/day | 2969.08 | +58.32 | 0.66 | +119.74 | 0.37 | +184.53 | 0.16 | +252.95 | 0.06 | +340.68 | 0.01* | +346.49 | 0.01* | +307.13 | 0.02* | +106.94 | 0.42 | -137.89 | 0.30 | +550.20 | <0.01* | +535.41 | <0.01* | +400.58 | <0.01* |
| Iron, mg/day | 14.42 | -0.07 | 0.91 | -0.14 | 0.81 | -0.22 | 0.72 | -0.30 | 0.62 | -0.53 | 0.38 | -1.07 | 0.07 | -0.43 | 0.47 | +8.47 | <0.01* | -1.01 | 0.09 | -0.04 | 0.94 | +3.46 | <0.01* | -0.60 | 0.32 |
| B12, ug/day | 6.93 | -0.44 | 0.31 | -0.91 | 0.04* | -1.40 | <0.01* | -1.92 | <0.01* | -1.94 | <0.01* | -1.65 | <0.01* | -1.89 | <0.01* | -2.24 | <0.01* | -2.09 | <0.01* | -1.96 | <0.01* | -0.37 | 0.40 | -1.99 | <0.01* |
| **Blue indicates significant increase compared to Current. Gold indicates significant decrease compared to Current.** Differences compared using regression models with significance threshold P < 0.05  MA-25, Replacement model substituting 25% of self-reported meat intake with composite of meat alternatives; MA-50, Replacement model substituting 50% of self-reported meat intake with composite of meat alternatives; MA-75, Replacement model substituting 75% of self-reported meat intake with composite of meat alternatives; MA-100, Replacement model substituting 100% of self-reported meat intake with composite of meat alternatives. | | | | | | | | | | | | | | | | | | | | | | | | | |

| **Supplementary Table 9.**  **Mean nutrient intake for current and replacement models, with reference to DRVs; females aged 11-18 years (n = 115)** | | | | | | | | | | | | | | |
| --- | --- | --- | --- | --- | --- | --- | --- | --- | --- | --- | --- | --- | --- | --- |
|  |  |  | **Progressive Model** | | | | **Ingredient Model** | | | | | | **Fortification Model** | |
| **Nutrient** | **DRV*** | **Current** | **MA-25** | **MA-50** | **MA-75** | **MA-100** | **Vegetable** | **Mycoprotein** | **Legume** | **Tofu** | **Nut** | **Soy** | **Fortified** | **Unfortified** |
| Energy, kcal/day | 2304 | - | - | - | - | - | - | - | - | - | - | - | - | - |
| Carbohydrate, g/day† | 287.90 | 304.27 | 308.63 | 313.22 | 318.07 | 323.18 | 328.73 | 326.53 | 324.06 | 313.21 | 315.67 | 318.74 | 315.43 | 327.38 |
| Protein,  g/day† | 86.40 | 89.78 | 86.40 | 82.84 | 79.08 | 75.11 | 70.58 | 81.02 | 74.45 | 84.94 | 70.12 | 82.64 | 86.71 | 71.72 |
| Fat,  g/day | 89.60 | 88.01 | 87.24 | 86.42 | 85.57 | 84.66 | 83.65 | 81.93 | 84.54 | 85.66 | 89.38 | 83.33 | 83.15 | 83.85 |
| Fibre,  g/day | 30.00 | 21.90 | 23.77 | 25.73 | 27.80 | 29.98 | 30.57 | 30.55 | 28.65 | 25.02 | 32.07 | 28.11 | 28.94 | 30.21 |
| Sugars,  g/day | 90.00 | 116.35 | 117.31 | 118.31 | 119.37 | 120.49 | 120.75 | 119.11 | 119.75 | 118.49 | 123.74 | 118.53 | 118.03 | 120.44 |
| Saturated fat, g/day | 28.20 | 31.66 | 30.75 | 29.79 | 28.78 | 27.72 | 27.85 | 28.03 | 27.95 | 27.36 | 26.89 | 27.99 | 28.38 | 27.74 |
| Sodium, mg/day | 2400 | 2601.91 | 2654.14 | 2709.14 | 2767.14 | 2828.41 | 2906.96 | 2912.17 | 2876.93 | 2697.68 | 2478.45 | 3094.57 | 3081.31 | 2960.59 |
| Iron,  mg/day | 14.80 | 14.72 | 14.66 | 14.59 | 14.52 | 14.45 | 14.24 | 13.76 | 14.33 | 22.30 | 13.82 | 14.68 | 17.82 | 14.18 |
| B12,  ug/day | 1.5 | 5.19 | 4.79 | 4.38 | 3.94 | 3.47 | 3.45 | 3.71 | 3.50 | 3.18 | 3.32 | 3.44 | 4.86 | 3.41 |
| *DRV values correspond to EAR for energy; RNI for Protein, Sodium, Iron, B12; DRV for Carbohydrate, Fat, Fibre, Sugars, Saturated fat.  † Protein target elected as 15% total energy intake and carbohydrate as 50% total energy intake in the current analysis.  Colours: Green, meeting DRV. Red, not meeting DRV. Grey, not meeting 15% protein and/or 50% carbohydrate target elected in the current analysis.  DRV, Dietary Reference Value; EAR, Estimated Average Requirement; RNI, Reference Nutrient Intake; MA-25, Replacement model substituting 25% of self-reported meat intake with composite of meat alternatives; MA-50, Replacement model substituting 50% of self-reported meat intake with composite of meat alternatives; MA-75, Replacement model substituting 75% of self-reported meat intake with composite of meat alternatives; MA-100, Replacement model substituting 100% of self-reported meat intake with composite of meat alternatives. | | | | | | | | | | | | | | |

| **Supplementary Table 10.  Difference in nutrient intake between replacement models and current intake; females aged 11-18 years (n = 115)** | | | | | | | | | | | | | | | | | | | | | | | | | |
| --- | --- | --- | --- | --- | --- | --- | --- | --- | --- | --- | --- | --- | --- | --- | --- | --- | --- | --- | --- | --- | --- | --- | --- | --- | --- |
|  |  | **Progressive Model** | | | | | | | | **Ingredient Model** | | | | | | | | | | | | **Fortification Model** | | | |
| **Nutrient** | **Current** | **MA-25** | **P** | **MA-50** | **P** | **MA-75** | **P** | **MA-100** | **P** | **Vegetable** | **P** | **Mycoprotein** | **P** | **Legume** | **P** | **Tofu** | **P** | **Nut** | **P** | **Soy** | **P** | **Fortified** | **P** | **Unfortified** | **P** |
| Energy, kcal/day | 2304.00 | - | - | - | - | - | - | - | - |  |  | - | - | - | - |  |  |  |  | - | - | - | - | - | - |
| Carbohydrate, g/day | 304.27 | +4.36 | 0.73 | +8.95 | 0.48 | +13.79 | 0.28 | +18.91 | 0.14 | +24.46 | 0.05* | +22.25 | 0.08 | +19.79 | 0.12 | +8.94 | 0.48 | +11.40 | 0.37 | +14.46 | 0.26 | +11.16 | 0.38 | +23.10 | 0.07 |
| Protein, g/day | 89.78 | -3.38 | 0.27 | -6.94 | 0.02* | -10.70 | <0.01* | -14.67 | <0.01* | -19.20 | <0.01* | -8.76 | <0.01* | -15.33 | <0.01* | -4.84 | 0.12 | -19.66 | <0.01* | -7.14 | 0.02* | -3.07 | 0.32 | -18.06 | <0.01* |
| Fat, g/day | 88.01 | -0.77 | 0.85 | -1.58 | 0.70 | -2.44 | 0.56 | -3.35 | 0.42 | -4.36 | 0.29 | -6.08 | 0.14 | -3.47 | 0.40 | -2.35 | 0.57 | +1.37 | 0.74 | -4.67 | 0.26 | -4.86 | 0.24 | -4.16 | 0.32 |
| Fibre, g/day | 21.90 | +1.86 | 0.07 | +3.82 | <0.01* | +5.89 | <0.01* | +8.08 | <0.01* | +8.67 | <0.01* | +8.65 | <0.01* | +6.75 | <0.01* | +3.12 | <0.01* | +10.17 | <0.01* | +6.21 | <0.01* | +7.03 | <0.01* | +8.31 | <0.01* |
| Sugars, g/day | 116.35 | +0.95 | 0.90 | +1.96 | 0.79 | +3.02 | 0.69 | +4.14 | 0.58 | +4.40 | 0.56 | +2.76 | 0.71 | +3.39 | 0.65 | +2.13 | 0.78 | +7.39 | 0.32 | +2.18 | 0.77 | +1.67 | 0.82 | +4.09 | 0.58 |
| Saturated fat, g/day | 31.66 | -0.91 | 0.61 | -1.86 | 0.29 | -2.87 | 0.11 | -3.94 | 0.03* | -3.80 | 0.03* | -3.63 | 0.04* | -3.70 | 0.04* | -4.30 | 0.02* | -4.77 | 0.01* | -3.66 | 0.04* | -3.28 | 0.07 | -3.91 | 0.03* |
| Sodium, mg/day | 2601.91 | +52.23 | 0.68 | +107.23 | 0.40 | +165.23 | 0.20 | +226.49 | 0.08 | +305.05 | 0.02* | +310.25 | 0.02* | +275.02 | 0.03* | +95.76 | 0.45 | -123.47 | 0.33 | +492.65 | <0.01* | +479.39 | <0.01* | +358.67 | 0.01* |
| Iron, mg/day | 14.72 | -0.06 | 0.99 | -0.13 | 0.97 | -0.19 | 0.96 | -0.27 | 0.94 | -0.47 | 0.90 | -0.96 | 0.80 | -0.39 | 0.92 | +7.58 | 0.05* | -0.90 | 0.81 | -0.04 | 0.99 | 3.10 | 0.41 | -0.54 | 0.89 |
| B12, ug/day | 5.19 | -0.40 | 0.18 | -0.81 | 0.01* | -1.25 | <0.01* | -1.72 | <0.01* | -1.74 | <0.01* | -1.48 | <0.01* | -1.69 | <0.01* | -2.01 | <0.01* | -1.87 | <0.01* | -1.75 | <0.01* | -0.33 | 0.26 | -1.78 | <0.01* |
| **Blue indicates significant increase compared to Current. Gold indicates significant decrease compared to Current.** Differences compared using regression models with significance threshold P < 0.05  MA-25, Replacement model substituting 25% of self-reported meat intake with composite of meat alternatives; MA-50, Replacement model substituting 50% of self-reported meat intake with composite of meat alternatives; MA-75, Replacement model substituting 75% of self-reported meat intake with composite of meat alternatives; MA-100, Replacement model substituting 100% of self-reported meat intake with composite of meat alternatives. | | | | | | | | | | | | | | | | | | | | | | | | | |

| **Supplementary Table 11.**  **Mean nutrient intake for current and replacement models, with reference to DRVs; males aged 19-64 years (n = 198)** | | | | | | | | | | | | | | |
| --- | --- | --- | --- | --- | --- | --- | --- | --- | --- | --- | --- | --- | --- | --- |
|  |  |  | **Progressive Model** | | | | **Ingredient Model** | | | | | | **Fortification Model** | |
| **Nutrient** | **DRV*** | **Current** | **MA-25** | **MA-50** | **MA-75** | **MA-100** | **Vegetable** | **Mycoprotein** | **Legume** | **Tofu** | **Nut** | **Soy** | **Fortified** | **Unfortified** |
| Energy, kcal/day | 2662 | - | - | - | - | - | - | - | - | - | - | - | - | - |
| Carbohydrate, g/day† | 332.80 | 317.53 | 323.43 | 329.63 | 336.18 | 343.09 | 350.59 | 347.61 | 344.27 | 329.62 | 332.94 | 337.08 | 332.62 | 348.76 |
| Protein,  g/day† | 99.80 | 112.20 | 107.63 | 102.81 | 97.73 | 92.37 | 86.24 | 100.36 | 91.48 | 105.66 | 85.63 | 102.54 | 108.04 | 87.78 |
| Fat,  g/day | 103.50 | 99.68 | 98.64 | 97.54 | 96.38 | 95.16 | 93.79 | 91.46 | 95.00 | 96.51 | 101.53 | 93.37 | 93.11 | 94.06 |
| Fibre,  g/day | 30.00 | 27.25 | 29.77 | 32.42 | 35.22 | 38.17 | 38.97 | 38.94 | 36.37 | 31.47 | 40.99 | 35.65 | 36.76 | 38.49 |
| Sugars,  g/day | 90.00 | 126.83 | 128.12 | 129.47 | 130.91 | 132.42 | 132.77 | 130.56 | 131.42 | 129.71 | 136.82 | 129.78 | 129.09 | 132.35 |
| Saturated fat, g/day | 32.50 | 36.64 | 35.41 | 34.12 | 32.75 | 31.31 | 31.50 | 31.73 | 31.63 | 30.83 | 30.20 | 31.69 | 32.21 | 31.35 |
| Sodium, mg/day | 2400 | 3114.72 | 3185.31 | 3259.64 | 3338.03 | 3420.85 | 3526.99 | 3534.03 | 3486.41 | 3244.14 | 2947.85 | 3780.55 | 3762.67 | 3599.48 |
| Iron,  mg/day | 8.70 | 16.05 | 15.96 | 15.88 | 15.78 | 15.69 | 15.41 | 14.75 | 15.52 | 26.30 | 14.83 | 15.99 | 20.23 | 15.32 |
| B12,  ug/day | 1.5 | 8.98 | 8.45 | 7.88 | 7.29 | 6.66 | 6.63 | 6.99 | 6.69 | 6.27 | 6.45 | 6.61 | 8.54 | 6.58 |
| *DRV values correspond to EAR for energy; RNI for Protein, Sodium, Iron, B12; DRV for Carbohydrate, Fat, Fibre, Sugars, Saturated fat.  † Protein target elected as 15% total energy intake and carbohydrate as 50% total energy intake in the current analysis.  Colours: Green, meeting DRV. Red, not meeting DRV. Grey, not meeting 15% protein and/or 50% carbohydrate target elected in the current analysis.  DRV, Dietary Reference Value; EAR, Estimated Average Requirement; RNI, Reference Nutrient Intake; MA-25, Replacement model substituting 25% of self-reported meat intake with composite of meat alternatives; MA-50, Replacement model substituting 50% of self-reported meat intake with composite of meat alternatives; MA-75, Replacement model substituting 75% of self-reported meat intake with composite of meat alternatives; MA-100, Replacement model substituting 100% of self-reported meat intake with composite of meat alternatives. | | | | | | | | | | | | | | |

| **Supplementary Table 12.  Difference in nutrient intake between replacement models and current intake; males aged 19-64 years (n = 198)** | | | | | | | | | | | | | | | | | | | | | | | | | |
| --- | --- | --- | --- | --- | --- | --- | --- | --- | --- | --- | --- | --- | --- | --- | --- | --- | --- | --- | --- | --- | --- | --- | --- | --- | --- |
|  |  | **Progressive Model** | | | | | | | | **Ingredient Model** | | | | | | | | | | | | **Fortification Model** | | | |
| **Nutrient** | **Current** | **MA-25** | **P** | **MA-50** | **P** | **MA-75** | **P** | **MA-100** | **P** | **Vegetable** | **P** | **Mycoprotein** | **P** | **Legume** | **P** | **Tofu** | **P** | **Nut** | **P** | **Soy** | **P** | **Fortified** | **P** | **Unfortified** | **P** |
| Energy, kcal/day | 2662.00 | - | - | - | - | - | - | - | - |  |  | - | - | - | - |  |  |  |  | - | - | - | - | - | - |
| Carbohydrate, g/day | 317.53 | +5.89 | 0.60 | +12.10 | 0.29 | +18.64 | 0.10 | +25.56 | 0.02* | +33.06 | <0.01* | +30.08 | 0.01* | +26.74 | 0.02* | +12.09 | 0.29 | +15.41 | 0.18 | +19.55 | 0.09 | +15.09 | 0.19 | +31.23 | 0.01* |
| Protein, g/day | 112.20 | -4.57 | 0.11 | -9.39 | <0.01* | -14.46 | <0.01* | -19.83 | <0.01* | -25.96 | <0.01* | -11.83 | <0.01* | -20.72 | <0.01* | -6.54 | 0.02* | -26.57 | <0.01* | -9.66 | <0.01* | -4.15 | 0.14 | -24.42 | <0.01* |
| Fat, g/day | 99.68 | -1.04 | 0.77 | -2.14 | 0.54 | -3.30 | 0.35 | -4.52 | 0.20 | -5.89 | 0.10 | -8.22 | 0.02* | -4.69 | 0.18 | -3.18 | 0.37 | +1.85 | 0.60 | -6.32 | 0.07 | -6.57 | 0.06 | -5.62 | 0.11 |
| Fibre, g/day | 27.25 | +2.52 | 0.04* | +5.17 | <0.01* | +7.97 | <0.01* | +10.92 | <0.01* | +11.71 | <0.01* | +11.69 | <0.01* | +9.12 | <0.01* | +4.21 | <0.01* | +13.74 | <0.01* | +8.39 | <0.01* | +9.51 | <0.01* | +11.23 | <0.01* |
| Sugars, g/day | 126.83 | +1.29 | 0.84 | +2.65 | 0.68 | +4.08 | 0.53 | +5.59 | 0.39 | +5.94 | 0.36 | +3.73 | 0.57 | +4.59 | 0.48 | +2.88 | 0.66 | +9.99 | 0.12 | +2.95 | 0.65 | +2.26 | 0.73 | +5.53 | 0.40 |
| Saturated fat, g/day | 36.64 | -1.23 | 0.42 | -2.52 | 0.10 | -3.88 | 0.01* | -5.32 | <0.01* | -5.14 | <0.01* | -4.90 | <0.01* | -5.01 | <0.01* | -5.81 | <0.01* | -6.44 | <0.01* | -4.95 | <0.01* | -4.43 | <0.01* | -5.29 | <0.01* |
| Sodium, mg/day | 3114.72 | +70.58 | 0.60 | +144.91 | 0.28 | +223.31 | 0.10 | +306.13 | 0.02* | +412.26 | <0.01* | +419.30 | <0.01* | +371.68 | 0.01* | +129.42 | 0.34 | -166.87 | 0.21 | +665.82 | <0.01* | +647.95 | <0.01* | +484.75 | <0.01* |
| Iron, mg/day | 16.05 | -0.08 | 0.90 | -0.17 | 0.79 | -0.26 | 0.69 | -0.36 | 0.58 | -0.64 | 0.33 | -1.30 | 0.05* | -0.52 | 0.42 | +10.25 | <0.01* | -1.22 | 0.06 | -0.05 | 0.93 | +4.19 | <0.01* | -0.73 | 0.26 |
| B12, ug/day | 8.98 | -0.54 | 0.65 | -1.10 | 0.35 | -1.69 | 0.15 | -2.32 | 0.05* | -2.35 | 0.05* | -2.00 | 0.09 | -2.29 | 0.05* | -2.72 | 0.02* | -2.53 | 0.03* | -2.37 | 0.04* | -0.44 | 0.71 | -2.40 | 0.04* |
| **Blue indicates significant increase compared to Current. Gold indicates significant decrease compared to Current.** Differences compared using regression models with significance threshold P < 0.05  MA-25, Replacement model substituting 25% of self-reported meat intake with composite of meat alternatives; MA-50, Replacement model substituting 50% of self-reported meat intake with composite of meat alternatives; MA-75, Replacement model substituting 75% of self-reported meat intake with composite of meat alternatives; MA-100, Replacement model substituting 100% of self-reported meat intake with composite of meat alternatives. | | | | | | | | | | | | | | | | | | | | | | | | | |

| **Supplementary Table 13.**  **Mean nutrient intake for current and replacement models, with reference to DRVs; females aged 19-64 years (n = 257)** | | | | | | | | | | | | | | |
| --- | --- | --- | --- | --- | --- | --- | --- | --- | --- | --- | --- | --- | --- | --- |
|  |  |  | **Progressive Model** | | | | **Ingredient Model** | | | | | | **Fortification Model** | |
| **Nutrient** | **DRV*** | **Current** | **MA-25** | **MA-50** | **MA-75** | **MA-100** | **Vegetable** | **Mycoprotein** | **Legume** | **Tofu** | **Nut** | **Soy** | **Fortified** | **Unfortified** |
| Energy, kcal/day | 2127 | - | - | - | - | - | - | - | - | - | - | - | - | - |
| Carbohydrate, g/day† | 265.90 | 257.49 | 261.74 | 266.21 | 270.92 | 275.90 | 281.30 | 279.15 | 276.75 | 266.20 | 268.59 | 271.57 | 268.36 | 279.98 |
| Protein,  g/day† | 79.80 | 90.18 | 86.89 | 83.42 | 79.76 | 75.90 | 71.49 | 81.66 | 75.26 | 85.47 | 71.05 | 83.23 | 87.19 | 72.60 |
| Fat,  g/day | 82.70 | 81.92 | 81.17 | 80.38 | 79.54 | 78.66 | 77.68 | 76.00 | 78.54 | 79.63 | 83.25 | 77.37 | 77.19 | 77.87 |
| Fibre,  g/day | 30.00 | 23.50 | 25.32 | 27.23 | 29.24 | 31.37 | 31.94 | 31.92 | 30.07 | 26.54 | 33.40 | 29.55 | 30.35 | 31.59 |
| Sugars,  g/day | 90.00 | 104.22 | 105.15 | 106.12 | 107.15 | 108.24 | 108.50 | 106.90 | 107.52 | 106.29 | 111.41 | 106.34 | 105.85 | 108.20 |
| Saturated fat, g/day | 26.00 | 30.33 | 29.45 | 28.52 | 27.54 | 26.50 | 26.63 | 26.80 | 26.73 | 26.15 | 25.70 | 26.77 | 27.14 | 26.53 |
| Sodium, mg/day | 2400 | 2416.49 | 2467.30 | 2520.83 | 2577.28 | 2636.91 | 2713.34 | 2718.42 | 2684.12 | 2509.67 | 2296.33 | 2895.91 | 2883.03 | 2765.54 |
| Iron,  mg/day | 14.80 | 15.93 | 15.87 | 15.80 | 15.74 | 15.67 | 15.47 | 14.99 | 15.55 | 23.31 | 15.05 | 15.89 | 18.94 | 15.40 |
| B12,  ug/day | 1.5 | 10.85 | 10.46 | 10.06 | 9.63 | 9.18 | 9.16 | 9.41 | 9.20 | 8.89 | 9.03 | 9.14 | 10.53 | 9.12 |
| *DRV values correspond to EAR for energy; RNI for Protein, Sodium, Iron, B12; DRV for Carbohydrate, Fat, Fibre, Sugars, Saturated fat.  † Protein target elected as 15% total energy intake and carbohydrate as 50% total energy intake in the current analysis.  Colours: Green, meeting DRV. Red, not meeting DRV. Grey, not meeting 15% protein and/or 50% carbohydrate target elected in the current analysis.  DRV, Dietary Reference Value; EAR, Estimated Average Requirement; RNI, Reference Nutrient Intake; MA-25, Replacement model substituting 25% of self-reported meat intake with composite of meat alternatives; MA-50, Replacement model substituting 50% of self-reported meat intake with composite of meat alternatives; MA-75, Replacement model substituting 75% of self-reported meat intake with composite of meat alternatives; MA-100, Replacement model substituting 100% of self-reported meat intake with composite of meat alternatives. | | | | | | | | | | | | | | |

| **Supplementary Table 14.  Difference in nutrient intake between replacement models and current intake; females aged 19-64 years (n = 257)** | | | | | | | | | | | | | | | | | | | | | | | | | |
| --- | --- | --- | --- | --- | --- | --- | --- | --- | --- | --- | --- | --- | --- | --- | --- | --- | --- | --- | --- | --- | --- | --- | --- | --- | --- |
|  |  | **Progressive Model** | | | | | | | | **Ingredient Model** | | | | | | | | | | | | **Fortification Model** | | | |
| **Nutrient** | **Current** | **MA-25** | **P** | **MA-50** | **P** | **MA-75** | **P** | **MA-100** | **P** | **Vegetable** | **P** | **Mycoprotein** | **P** | **Legume** | **P** | **Tofu** | **P** | **Nut** | **P** | **Soy** | **P** | **Fortified** | **P** | **Unfortified** | **P** |
| Energy, kcal/day | 2127.00 | - | - | - | - | - | - | - | - |  |  | - | - | - | - |  |  |  |  | - | - | - | - | - | - |
| Carbohydrate, g/day | 257.49 | +4.24 | 0.59 | +8.71 | 0.27 | +13.42 | 0.09 | +18.40 | 0.02* | +23.81 | <0.01* | +21.66 | 0.01* | +19.25 | 0.02* | +8.70 | 0.27 | +11.09 | 0.16 | +14.08 | 0.08 | +10.86 | 0.17 | +22.48 | <0.01* |
| Protein, g/day | 90.18 | -3.29 | 0.14 | -6.76 | <0.01* | -10.41 | <0.01* | -14.28 | <0.01* | -18.69 | <0.01* | -8.52 | <0.01* | -14.92 | <0.01* | -4.71 | 0.04* | -19.13 | <0.01* | -6.95 | <0.01* | -2.99 | 0.18 | -17.58 | <0.01* |
| Fat, g/day | 81.92 | -0.75 | 0.79 | -1.54 | 0.59 | -2.38 | 0.41 | -3.26 | 0.25 | -4.24 | 0.14 | -5.92 | 0.04* | -3.37 | 0.24 | -2.29 | 0.42 | +1.33 | 0.64 | -4.55 | 0.11 | -4.73 | 0.10 | -4.05 | 0.16 |
| Fibre, g/day | 23.50 | +1.81 | 0.05* | +3.72 | <0.01* | +5.74 | <0.01* | +7.86 | <0.01* | +8.44 | <0.01* | +8.42 | <0.01* | +6.57 | <0.01* | +3.03 | <0.01* | +9.89 | <0.01* | +6.04 | <0.01* | +6.85 | <0.01* | +8.09 | <0.01* |
| Sugars, g/day | 104.22 | +0.93 | 0.84 | +1.91 | 0.69 | +2.94 | 0.53 | +4.03 | 0.39 | +4.28 | 0.36 | +2.68 | 0.57 | +3.30 | 0.48 | +2.08 | 0.66 | +7.19 | 0.13 | +2.12 | 0.65 | +1.63 | 0.73 | +3.98 | 0.40 |
| Saturated fat, g/day | 30.33 | -0.88 | 0.46 | -1.81 | 0.13 | -2.80 | 0.02* | -3.83 | <0.01* | -3.70 | <0.01* | -3.53 | <0.01* | -3.60 | <0.01* | -4.18 | <0.01* | -4.64 | <0.01* | -3.57 | <0.01* | -3.19 | 0.01* | -3.81 | <0.01* |
| Sodium, mg/day | 2416.49 | +50.81 | 0.51 | +104.34 | 0.18 | +160.79 | 0.04* | +220.43 | <0.01* | +296.85 | <0.01* | +301.93 | <0.01* | +267.64 | <0.01* | +93.18 | 0.23 | -120.15 | 0.12 | +479.42 | <0.01* | +466.54 | <0.01* | +349.05 | <0.01* |
| Iron, mg/day | 15.93 | -0.06 | 0.98 | -0.12 | 0.95 | -0.19 | 0.92 | -0.26 | 0.90 | -0.46 | 0.82 | -0.94 | 0.64 | -0.38 | 0.85 | +7.38 | <0.01* | -0.88 | 0.66 | -0.04 | 0.98 | +3.01 | 0.13 | -0.53 | 0.79 |
| B12, ug/day | 10.85 | -0.39 | 0.92 | -0.79 | 0.84 | -1.22 | 0.76 | -1.67 | 0.67 | -1.69 | 0.67 | -1.44 | 0.72 | -1.65 | 0.68 | -1.96 | 0.62 | -1.82 | 0.65 | -1.71 | 0.67 | -0.32 | 0.94 | -1.73 | 0.66 |
| **Blue indicates significant increase compared to Current. Gold indicates significant decrease compared to Current.** Differences compared using regression models with significance threshold P < 0.05  MA-25, Replacement model substituting 25% of self-reported meat intake with composite of meat alternatives; MA-50, Replacement model substituting 50% of self-reported meat intake with composite of meat alternatives; MA-75, Replacement model substituting 75% of self-reported meat intake with composite of meat alternatives; MA-100, Replacement model substituting 100% of self-reported meat intake with composite of meat alternatives. | | | | | | | | | | | | | | | | | | | | | | | | | |

| **Supplementary Table 15.**  **Mean nutrient intake for current and replacement models, with reference to DRVs; males aged ≤ 65 years (n = 66)** | | | | | | | | | | | | | | |
| --- | --- | --- | --- | --- | --- | --- | --- | --- | --- | --- | --- | --- | --- | --- |
|  |  |  | **Progressive Model** | | | | **Ingredient Model** | | | | | | **Fortification Model** | |
| **Nutrient** | **DRV*** | **Current** | **MA-25** | **MA-50** | **MA-75** | **MA-100** | **Vegetable** | **Mycoprotein** | **Legume** | **Tofu** | **Nut** | **Soy** | **Fortified** | **Unfortified** |
| Energy, kcal/day | 2318 | - | - | - | - | - | - | - | - | - | - | - | - | - |
| Carbohydrate, g/day | 289.80 | 263.86 | 268.87 | 274.15 | 279.72 | 285.60 | 291.98 | 289.44 | 286.60 | 274.14 | 276.97 | 280.49 | 276.69 | 290.42 |
| Protein,  g/day | 86.90 | 94.26 | 90.37 | 86.27 | 81.96 | 77.39 | 72.18 | 84.19 | 76.63 | 88.70 | 71.66 | 86.04 | 90.72 | 73.49 |
| Fat,  g/day | 90.10 | 89.56 | 88.68 | 87.74 | 86.76 | 85.72 | 84.56 | 82.57 | 85.58 | 86.86 | 91.14 | 84.19 | 83.98 | 84.78 |
| Fibre,  g/day | 30.00 | 24.15 | 26.29 | 28.55 | 30.93 | 33.44 | 34.12 | 34.10 | 31.91 | 27.74 | 35.84 | 31.29 | 32.24 | 33.71 |
| Sugars,  g/day | 90.00 | 115.14 | 116.23 | 117.39 | 118.61 | 119.89 | 120.19 | 118.31 | 119.04 | 117.59 | 123.64 | 117.65 | 117.06 | 119.84 |
| Saturated fat, g/day | 28.30 | 33.45 | 32.41 | 31.31 | 30.15 | 28.93 | 29.08 | 29.28 | 29.20 | 28.51 | 27.98 | 29.24 | 29.69 | 28.96 |
| Sodium, mg/day | 2400 | 2460.51 | 2520.54 | 2583.76 | 2650.44 | 2720.88 | 2811.16 | 2817.13 | 2776.64 | 2570.59 | 2318.58 | 3026.83 | 3011.61 | 2872.80 |
| Iron,  mg/day | 8.70 | 15.10 | 15.03 | 14.95 | 14.87 | 14.79 | 14.55 | 13.99 | 14.65 | 23.81 | 14.06 | 15.05 | 18.66 | 14.48 |
| B12,  ug/day | 1.5 | 8.41 | 7.95 | 7.47 | 6.97 | 6.44 | 6.41 | 6.71 | 6.46 | 6.10 | 6.26 | 6.39 | 8.03 | 6.37 |
| *DRV values correspond to EAR for energy; RNI for Protein, Sodium, Iron, B12; DRV for Carbohydrate, Fat, Fibre, Sugars, Saturated fat.  † Protein target elected as 15% total energy intake and carbohydrate as 50% total energy intake in the current analysis.  Colours: Green, meeting DRV. Red, not meeting DRV. Grey, not meeting 15% protein and/or 50% carbohydrate target elected in the current analysis.  DRV, Dietary Reference Value; EAR, Estimated Average Requirement; RNI, Reference Nutrient Intake; MA-25, Replacement model substituting 25% of self-reported meat intake with composite of meat alternatives; MA-50, Replacement model substituting 50% of self-reported meat intake with composite of meat alternatives; MA-75, Replacement model substituting 75% of self-reported meat intake with composite of meat alternatives; MA-100, Replacement model substituting 100% of self-reported meat intake with composite of meat alternatives. | | | | | | | | | | | | | | |

| **Supplementary Table 16.  Difference in nutrient intake between replacement models and current intake; males aged ≤ 65 years (n = 66)** | | | | | | | | | | | | | | | | | | | | | | | | | |
| --- | --- | --- | --- | --- | --- | --- | --- | --- | --- | --- | --- | --- | --- | --- | --- | --- | --- | --- | --- | --- | --- | --- | --- | --- | --- |
|  |  | **Progressive Model** | | | | | | | | **Ingredient Model** | | | | | | | | | | | | **Fortification Model** | | | |
| **Nutrient** | **Current** | **MA-25** | **P** | **MA-50** | **P** | **MA-75** | **P** | **MA-100** | **P** | **Vegetable** | **P** | **Mycoprotein** | **P** | **Legume** | **P** | **Tofu** | **P** | **Nut** | **P** | **Soy** | **P** | **Fortified** | **P** | **Unfortified** | **P** |
| Energy, kcal/day | 2318.00 | - | - | - | - | - | - | - | - |  |  | - | - | - | - |  |  |  |  | - | - | - | - | - | - |
| Carbohydrate, g/day | 263.86 | +5.01 | 0.66 | +10.29 | 0.37 | +15.86 | 0.17 | +21.74 | 0.06 | +28.12 | 0.01* | +25.58 | 0.03* | +22.74 | 0.05* | +10.28 | 0.37 | +9.20 | 0.20 | +16.63 | 0.15 | +12.83 | 0.26 | +26.56 | 0.02* |
| Protein, g/day | 94.26 | -3.89 | 0.26 | -7.98 | 0.02* | -12.30 | <0.01* | -16.86 | <0.01* | -22.08 | <0.01* | -10.07 | <0.01* | -17.62 | <0.01* | -5.56 | 0.11 | -22.60 | <0.01* | -8.21 | 0.02* | -3.53 | 0.31 | -20.77 | <0.01* |
| Fat, g/day | 89.56 | -0.89 | 0.88 | -1.82 | 0.75 | -2.81 | 0.62 | -3.85 | 0.50 | -5.01 | 0.38 | -6.99 | 0.22 | -3.99 | 0.48 | -2.70 | 0.63 | +1.57 | 0.78 | -5.37 | 0.34 | -5.59 | 0.32 | -4.78 | 0.40 |
| Fibre, g/day | 24.15 | +2.14 | 0.16 | +4.40 | <0.01* | +6.77 | <0.01* | +9.29 | <0.01* | +9.96 | <0.01* | +9.94 | <0.01* | +7.76 | <0.01* | +3.58 | 0.02* | +11.69 | <0.01* | +7.14 | <0.01* | +8.09 | <0.01* | +9.55 | <0.01* |
| Sugars, g/day | 115.14 | +1.10 | 0.89 | +2.25 | 0.78 | +3.47 | 0.67 | +4.75 | 0.56 | +5.05 | 0.54 | +3.17 | 0.70 | +3.90 | 0.63 | +2.45 | 0.77 | +8.50 | 0.30 | +2.51 | 0.76 | +1.92 | 0.81 | +4.70 | 0.57 |
| Saturated fat, g/day | 33.45 | -1.04 | 0.66 | -2.14 | 0.37 | -3.30 | 0.17 | -4.53 | 0.06 | -4.37 | 0.07 | -4.17 | 0.08 | -4.26 | 0.07 | -4.94 | 0.04* | -5.48 | 0.02* | -4.21 | 0.08 | -3.77 | 0.11 | -4.50 | 0.06 |
| Sodium, mg/day | 2460.51 | +60.03 | 0.65 | +123.25 | 0.35 | +189.93 | 0.15 | +260.37 | 0.05* | +350.65 | 0.01* | +356.62 | 0.01* | +316.13 | 0.02* | +110.08 | 0.41 | -141.93 | 0.28 | +566.32 | <0.01* | +551.10 | <0.01* | +412.29 | <0.01* |
| Iron, mg/day | 15.10 | -0.07 | 0.97 | -0.15 | 0.95 | -0.22 | 0.92 | -0.31 | 0.89 | -0.55 | 0.80 | -1.11 | 0.61 | -0.45 | 0.83 | +8.72 | <0.01* | -1.04 | 0.63 | -0.05 | 0.98 | +3.56 | 0.10 | -0.62 | 0.77 |
| B12, ug/day | 8.41 | -0.46 | 0.56 | -0.93 | 0.23 | -1.44 | 0.06 | -1.97 | 0.01* | -2.00 | 0.01* | -1.70 | 0.03* | -1.95 | 0.01* | -2.31 | <0.01* | -2.15 | 0.01* | -2.01 | 0.01* | -0.38 | 0.63 | -2.04 | 0.01* |
| **Blue indicates significant increase compared to Current. Gold indicates significant decrease compared to Current.** Differences compared using regression models with significance threshold P < 0.05  MA-25, Replacement model substituting 25% of self-reported meat intake with composite of meat alternatives; MA-50, Replacement model substituting 50% of self-reported meat intake with composite of meat alternatives; MA-75, Replacement model substituting 75% of self-reported meat intake with composite of meat alternatives; MA-100, Replacement model substituting 100% of self-reported meat intake with composite of meat alternatives. | | | | | | | | | | | | | | | | | | | | | | | | | |

| **Supplementary Table 17.**  **Mean nutrient intake for current and replacement models, with reference to DRVs; females aged ≤ 65 years (n = 98)** | | | | | | | | | | | | | | |
| --- | --- | --- | --- | --- | --- | --- | --- | --- | --- | --- | --- | --- | --- | --- |
|  |  |  | **Progressive Model** | | | | **Ingredient Model** | | | | | | **Fortification Model** | |
| **Nutrient** | **DRV*** | **Current** | **MA-25** | **MA-50** | **MA-75** | **MA-100** | **Vegetable** | **Mycoprotein** | **Legume** | **Tofu** | **Nut** | **Soy** | **Fortified** | **Unfortified** |
| Energy, kcal/day | 1876 | - | - | - | - | - | - | - | - | - | - | - | - | - |
| Carbohydrate, g/day† | 234.50 | 226.87 | 230.32 | 233.95 | 237.78 | 241.83 | 246.23 | 244.48 | 242.53 | 233.94 | 235.89 | 238.31 | 235.70 | 245.15 |
| Protein ,  g/day† | 70.40 | 76.98 | 74.30 | 71.48 | 68.51 | 65.37 | 61.78 | 70.05 | 64.84 | 73.15 | 61.42 | 71.32 | 74.54 | 62.68 |
| Fat,  g/day | 73.00 | 74.96 | 74.35 | 73.71 | 73.03 | 72.31 | 71.51 | 70.15 | 72.21 | 73.10 | 76.04 | 71.26 | 71.11 | 71.67 |
| Fibre,  g/day | 30.00 | 21.05 | 22.53 | 24.08 | 25.72 | 27.45 | 27.91 | 27.90 | 26.39 | 23.52 | 29.10 | 25.97 | 26.62 | 27.63 |
| Sugars,  g/day | 90.00 | 100.74 | 101.50 | 102.29 | 103.13 | 104.02 | 104.22 | 102.93 | 103.43 | 102.43 | 106.59 | 102.47 | 102.07 | 103.98 |
| Saturated fat, g/day | 22.90 | 30.43 | 29.71 | 28.96 | 28.16 | 27.32 | 27.42 | 27.56 | 27.50 | 27.03 | 26.66 | 27.53 | 27.84 | 27.34 |
| Sodium, mg/day | 2400 | 2038.91 | 2080.24 | 2123.78 | 2169.69 | 2218.18 | 2280.33 | 2284.47 | 2256.57 | 2114.70 | 1941.19 | 2428.82 | 2418.35 | 2322.78 |
| Iron,  mg/day | 8.70 | 13.97 | 13.93 | 13.87 | 13.82 | 13.76 | 13.60 | 13.21 | 13.67 | 19.98 | 13.26 | 13.94 | 16.43 | 13.55 |
| B12,  ug/day | 1.5 | 6.72 | 6.41 | 6.08 | 5.73 | 5.36 | 5.34 | 5.55 | 5.38 | 5.13 | 5.24 | 5.33 | 6.46 | 5.31 |
| *DRV values correspond to EAR for energy; RNI for Protein, Sodium, Iron, B12; DRV for Carbohydrate, Fat, Fibre, Sugars, Saturated fat.  † Protein target elected as 15% total energy intake and carbohydrate as 50% total energy intake in the current analysis.  Colours: Green, meeting DRV. Red, not meeting DRV. Grey, not meeting 15% protein and/or 50% carbohydrate target elected in the current analysis.  DRV, Dietary Reference Value; EAR, Estimated Average Requirement; RNI, Reference Nutrient Intake; MA-25, Replacement model substituting 25% of self-reported meat intake with composite of meat alternatives; MA-50, Replacement model substituting 50% of self-reported meat intake with composite of meat alternatives; MA-75, Replacement model substituting 75% of self-reported meat intake with composite of meat alternatives; MA-100, Replacement model substituting 100% of self-reported meat intake with composite of meat alternatives. | | | | | | | | | | | | | | |

| **Supplementary Table 18.  Difference in nutrient intake between replacement models and current intake; females aged ≤ 65 years (n = 98)** | | | | | | | | | | | | | | | | | | | | | | | | | |
| --- | --- | --- | --- | --- | --- | --- | --- | --- | --- | --- | --- | --- | --- | --- | --- | --- | --- | --- | --- | --- | --- | --- | --- | --- | --- |
|  |  | **Progressive Model** | | | | | | | | **Ingredient Model** | | | | | | | | | | | | **Fortification Model** | | | |
| **Nutrient** | **Current** | **MA-25** | **P** | **MA-50** | **P** | **MA-75** | **P** | **MA-100** | **P** | **Vegetable** | **P** | **Mycoprotein** | **P** | **Legume** | **P** | **Tofu** | **P** | **Nut** | **P** | **Soy** | **P** | **Fortified** | **P** | **Unfortified** | **P** |
| Energy, kcal/day | 1876.00 | - | - | - | - | - | - | - | - |  |  | - | - | - | - |  |  |  |  | - | - | - | - | - | - |
| Carbohydrate, g/day | 226.87 | +3.45 | 0.69 | +7.09 | 0.42 | +10.92 | 0.21 | +14.97 | 0.09 | +19.36 | 0.03* | +17.61 | 0.05* | +15.66 | 0.07 | +7.08 | 0.42 | +9.02 | 0.30 | +11.45 | 0.19 | +8.84 | 0.31 | +18.29 | 0.04* |
| Protein, g/day | 76.98 | -2.68 | 0.31 | -5.50 | 0.04* | -8.47 | <0.01* | -11.61 | <0.01* | -15.20 | <0.01* | -6.93 | 0.01* | -12.13 | <0.01* | -3.83 | 0.15 | -15.56 | <0.01* | -5.65 | 0.03* | -2.43 | 0.36 | -14.30 | <0.01* |
| Fat, g/day | 74.96 | -0.61 | 0.86 | -1.25 | 0.71 | -1.97 | 0.42 | -2.65 | 0.44 | -3.45 | 0.31 | -4.81 | 0.16 | -2.74 | 0.42 | -1.86 | 0.59 | +1.08 | 0.75 | -3.70 | 0.28 | -3.85 | 0.26 | -3.29 | 0.34 |
| Fibre, g/day | 21.05 | +1.47 | 0.22 | +3.03 | 0.01* | +4.66 | <0.01* | +6.39 | <0.01* | +6.86 | <0.01* | +6.85 | <0.01* | +5.34 | <0.01* | +2.47 | 0.04* | +8.05 | <0.01* | +4.92 | <0.01* | +5.57 | <0.01* | +6.58 | <0.01* |
| Sugars, g/day | 100.74 | +0.75 | 0.90 | +1.55 | 0.79 | +2.39 | 0.69 | +3.27 | 0.58 | +3.48 | 0.56 | +2.18 | 0.71 | +2.69 | 0.65 | +1.72 | 0.64 | +5.85 | 0.32 | +1.73 | 0.77 | +1.32 | 0.82 | +3.24 | 0.59 |
| Saturated fat, g/day | 30.43 | -0.72 | 0.65 | -1.48 | 0.35 | -2.27 | 0.15 | -3.18 | <0.01* | -3.01 | 0.06 | -2.87 | 0.07 | -2.93 | 0.07 | -3.40 | 0.03* | -3.77 | 0.02* | -2.90 | 0.07 | -2.59 | 0.10 | -3.10 | 0.05* |
| Sodium, mg/day | 2038.91 | +41.33 | 0.63 | +84.87 | 0.33 | +130.78 | 0.13 | +179.27 | 0.04* | +241.42 | 0.01* | +245.56 | <0.01* | +217.66 | 0.01* | +75.80 | 0.38 | -97.72 | 0.26 | +389.91 | <0.01* | +379.44 | <0.01* | +283.87 | <0.01* |
| Iron, mg/day | 13.97 | -0.05 | 0.99 | -0.10 | 0.98 | -0.15 | 0.97 | -0.21 | 0.96 | -0.38 | 0.92 | -0.76 | 0.84 | -0.31 | 0.94 | +6.00 | 0.12 | -0.71 | 0.85 | -0.03 | 0.99 | +2.45 | 0.52 | -0.43 | 0.91 |
| B12, ug/day | 6.72 | -0.31 | 0.77 | -0.64 | 0.54 | -0.99 | 0.35 | -1.36 | 0.20 | -1.38 | 0.20 | -1.17 | 0.27 | -1.34 | 0.21 | -1.59 | 0.13 | -1.48 | 0.16 | -1.39 | 0.19 | -0.26 | 0.81 | -1.41 | 0.19 |
| **Blue indicates significant increase compared to Current. Gold indicates significant decrease compared to Current.** Differences compared using regression models with significance threshold P < 0.05  MA-25, Replacement model substituting 25% of self-reported meat intake with composite of meat alternatives; MA-50, Replacement model substituting 50% of self-reported meat intake with composite of meat alternatives; MA-75, Replacement model substituting 75% of self-reported meat intake with composite of meat alternatives; MA-100, Replacement model substituting 100% of self-reported meat intake with composite of meat alternatives. | | | | | | | | | | | | | | | | | | | | | | | | | |
